# Viral Escape from Neutralizing Antibodies in Early Subtype A HIV-1 Infection Drives an Increase in Autologous Neutralization Breadth

**DOI:** 10.1371/journal.ppat.1003173

**Published:** 2013-02-28

**Authors:** Megan K. Murphy, Ling Yue, Ruimin Pan, Saikat Boliar, Anurag Sethi, Jianhui Tian, Katja Pfafferot, Etienne Karita, Susan A. Allen, Emmanuel Cormier, Paul A. Goepfert, Persephone Borrow, James E. Robinson, S. Gnanakaran, Eric Hunter, Xiang-Peng Kong, Cynthia A. Derdeyn

**Affiliations:** 1 Immunology and Molecular Pathogenesis Graduate Program, Emory University, Atlanta, Georgia, United States of America; 2 Emory Vaccine Center at Yerkes National Primate Research Center, Emory University, Atlanta, Georgia, United States of America; 3 Department of Biochemistry and Molecular Pharmacology, New York University School of Medicine, New York, New York, United States of America; 4 Theoretical Biology and Biophysics Group, Los Alamos National Laboratory, Los Alamos, New Mexico, United States of America; 5 Nuffield Department of Clinical Medicine, University of Oxford, Oxford, United Kingdom; 6 Projet San Francisco, Kigali, Rwanda; 7 Department of Pathology and Laboratory Medicine, Emory University, Atlanta, Georgia, United States of America; 8 Departments of Epidemiology and Global Health, Emory University, Atlanta, Georgia, United States of America; 9 International AIDS Vaccine Initiative, Human Immunology Laboratory, Imperial College, London, United Kingdom; 10 Departments of Medicine and Microbiology, University of Alabama at Birmingham, Birmingham, Alabama, United States of America; 11 Department of Pediatrics, Tulane University School of Medicine, New Orleans, Louisiana, United States of America; University of Zurich, Switzerland

## Abstract

Antibodies that neutralize (nAbs) genetically diverse HIV-1 strains have been recovered from a subset of HIV-1 infected subjects during chronic infection. Exact mechanisms that expand the otherwise narrow neutralization capacity observed during early infection are, however, currently undefined. Here we characterized the earliest nAb responses in a subtype A HIV-1 infected Rwandan seroconverter who later developed moderate cross-clade nAb breadth, using (i) envelope (Env) glycoproteins from the transmitted/founder virus and twenty longitudinal nAb escape variants, (ii) longitudinal autologous plasma, and (iii) autologous monoclonal antibodies (mAbs). Initially, nAbs targeted a single region of gp120, which flanked the V3 domain and involved the alpha2 helix. A single amino acid change at one of three positions in this region conferred early escape. One immunoglobulin heavy chain and two light chains recovered from autologous B cells comprised two mAbs, 19.3H-L1 and 19.3H-L3, which neutralized the founder Env along with one or three of the early escape variants carrying these mutations, respectively. Neither mAb neutralized later nAb escape or heterologous Envs. Crystal structures of the antigen-binding fragments (Fabs) revealed flat epitope contact surfaces, where minimal light chain mutation in 19.3H-L3 allowed for additional antigenic interactions. Resistance to mAb neutralization arose in later Envs through alteration of two glycans spatially adjacent to the initial escape signatures. The cross-neutralizing nAbs that ultimately developed failed to target any of the defined V3-proximal changes generated during the first year of infection in this subject. Our data demonstrate that this subject's first recognized nAb epitope elicited strain-specific mAbs, which incrementally acquired autologous breadth, and directed later B cell responses to target distinct portions of Env. This immune re-focusing could have triggered the evolution of cross-clade antibodies and suggests that exposure to a specific sequence of immune escape variants might promote broad humoral responses during HIV-1 infection.

## Author Summary

Since cases were first recognized in the United States in 1981, human immunodeficiency virus (HIV-1) has infected over one million Americans. Globally, this scale reaches into the tens of millions, but no effective vaccine exists. Of those infected, approximately 20–30% of patients will develop broadly neutralizing antibodies. The reasons for maturation of these potentially protective responses are presently unknown, but being able to elicit such antibodies via vaccination could curb the pandemic. Here, we defined the earliest neutralizing antibody targets and the consequent routes of viral escape in one subtype A HIV-1 infected subject who developed modest breadth. We also determined the genetic and structural characteristics of early neutralizing monoclonal antibodies circulating in this subject and found that subtle light chain alteration enhanced target contact and neutralization. Overall, our data support the idea that exposure to a specific sequence of viral variants, which have escaped from immune pressure, could program long-term potential for antibody breadth.

## Introduction

Protective vaccines against viral infections generally elicit nAb responses that are comparable to those in natural infections [1]. It is, therefore, widely accepted that an optimal vaccine against HIV-1 will need to produce nAbs, but features such as the high genetic diversity and mutability of HIV-1 Env pose unique obstacles. While broad neutralization of HIV-1 will likely be difficult to achieve through immunization, renewed optimism exists because of breakthroughs in the HIV-1 vaccine and nAb research fields. In the recently concluded RV144 vaccine trial, modest protection from acquisition of infection was observed and correlated with high levels of antibodies that recognized the V1V2 hypervariable domain of Env gp120 [2]. To date, these anti-V1V2 antibodies are the only immune correlate of vaccine-mediated protection against HIV-1 in humans. In non-human primate models, Barouch *et al*. reported that strong vaccine-induced protection against a diverse simian immunodeficiency virus (SIV) challenge in rhesus macaques correlated with V2-binding antibody titer along with nAb titers against two neutralization-sensitive heterologous SIV Envs [3]. Taken together, these results support the concept that antibodies are important for protection against HIV-1 infection and lead to the hypothesis that even higher vaccine efficacy could be achieved if broad nAbs can be induced [4].

The latest intensified efforts to recover and characterize potent and broad mAbs from chronically infected subjects with exceptional neutralization breadth have yielded important clues regarding how these mAbs overcome Env diversity. Such cross-clade neutralizing mAbs have been shown to target conserved elements in the CD4 binding site (CD4bs) (e.g. VRC01, PGV04), V1V2-dependent and trimer-enhanced (quaternary) epitopes (e.g. PG9, PG16), the gp41 membrane proximal external region (MPER) (e.g. 4E10, CAP206, 10E8), and glycan/V3-dependent epitopes (e.g. PGT128) [5]–[11]. For each class of ‘super’ mAb, characterization of the variable domains of the immunoglobulin heavy and light chains (VH and VL, respectively), in terms of their structure, germline gene utilization, level of somatic hypermutation, and the features of their heavy chain third complementarity-determining regions (CDR H3s), has unveiled specific characteristics that facilitate extraordinary neutralizing capacity [8], [12]–[15]. Importantly, substantial nAb breadth usually requires two to three years of infection to develop and occurs in only about 20–30% of infected subjects [16], [17]. Furthermore, individuals with ‘elite’ neutralizing activity constitute only about 1% of chronically infected subjects [18]. The reasons why nAb breadth does not develop earlier or more frequently are not known but could include autoreactivity leading to clonal deletion of B cells [19], impaired affinity maturation [20], or induction of a particular Ig germline family [10], [13], [15], [21], [22]. It is also possible that early viral escape contributes to the process of increasing nAb breadth [23], [24].

A paradox of neutralization breadth is that targets known to mediate this phenomenon, such as HXB2 residue N160 in V2 (targeted by PG9, PG16) or N332 near V3 (targeted by PGT128), are well conserved and present in many transmitted/founder Envs, but broad cross-clade activity only develops in a subset of individuals. The mere presence of these targets is not, then, sufficient to elicit broadly neutralizing antibodies in early infection. Here we describe the initial nAbs in a subtype A HIV-1 infection that target the N332-proximal region of gp120 that has been previously associated with broad neutralization by mAbs recovered from a chronic subtype CRF02_AG infection [6], [8] and strain-specific nAb responses in early subtype B infection [25]. Early escape involved a single amino acid substitution in this region, which appeared to drive a modest increase in the autologous neutralization breadth of somatically related mAbs. Later escape entailed the addition and/or shifting of glycans recognized by several previously described broadly neutralizing mAbs, but these changes were not targeted by the cross-neutralizing nAbs that developed later in this subject. The combinatorial interplay among early nAb targets, viral escape pathways, and antibody somatic hypermutation could, therefore, shape the ultimate development of heterologous nAb breadth across subjects.

## Results

### Subtype A HIV-1 undergoes alternating cycles of antibody neutralization and viral escape during the first year of infection

To examine the course and magnitude of autologous HIV-specific humoral activity in a Rwandan seroconverter, R880F, establishment and evolution of the earliest detectable nAb responses were evaluated. This subject was identified as antigen positive, antibody negative on 5Jan07 and then as antibody positive on 12Jan07. This latter date of seroconversion was designated as the 0-month time point for our analyses. Subsequent samples were chronologically coded from this originating time point forward, and each Env was given an arbitrary letter (A, B, or C) and number (1–61) designation that was preceded by the time point of isolation in months post-seroconversion. For example, Envs 0-A6 and 0-B24 were singly isolated from 0-month plasma, 2-A9 and 2-A13 from 2-month plasma, 5-A5 and 5-B52 from 5-month plasma, etc. (Table 1). These viral Envs were single-genome amplified and cloned into expression plasmids for the evaluation of Env pseudotypes. The two 0-month Envs, 0-A6 and 0-B24, had identical sequences and represented the transmitted/founder virus (Figure S1). In sum, ten envelopes from plasma at 2-months post-seroconversion, three from 5-months, five from 7-months, and two from 10-months were evaluated (Table 1).

**Table 1:** Chronology of R880F envelope/monoclonal antibody isolation.

Sample Date	Time Point	HIV-1 Envelopes	Monoclonal Antibodies
12Jan07	0-month^a^	A6, B24	
16Mar07	2-month	A9, A13, B31, A23, A24, B18, A3, B1, B4, B12	
8Jun07	5-month	A5, B52, B53	
31Aug07	7-month	B1, B19, B27, B61, B45	
21Nov07	10-month	B39, C1	
19May08	16-month		19.3H-L1, 19.3H-L3

a Viral load at this time point was ∼580,422 copies/ml in plasma.

**Figure 1:**
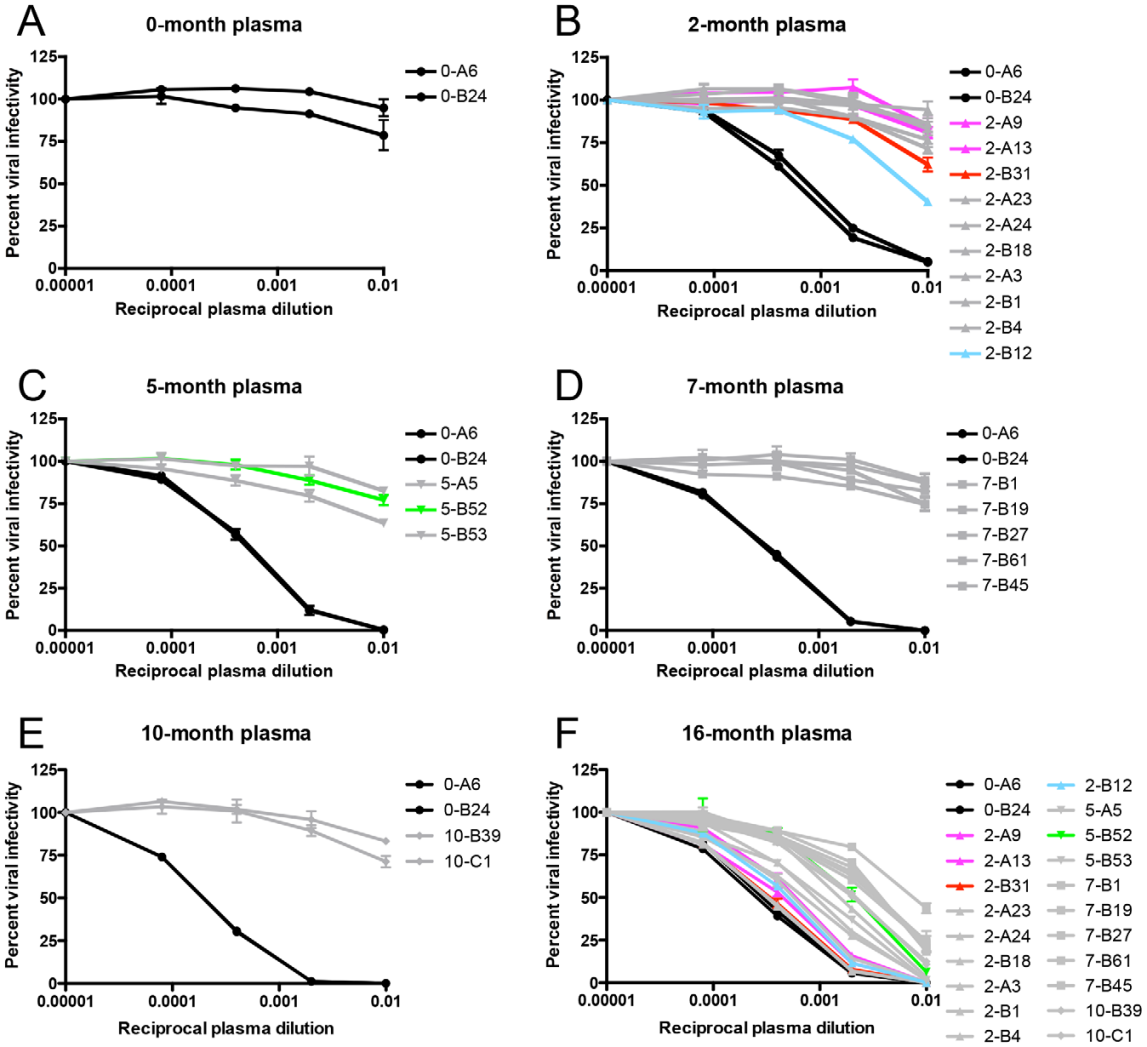
Identification of R880F nAb escape variants. Twenty-two single-genome amplified subtype A HIV-1 Envs were cloned out of R880F plasma collected at 0-months (**A**), 2-months (**B**), 5-months (**C**), 7-months (**D**), and 10-months (**E**) post-seroconversion, pseudotyped, and assayed against autologous plasma contemporaneous to their respective dates of isolation. Two 0-month Envs (0-A6/B24) representative of the transmitted/founder sequence are included in each panel. To demonstrate that humoral escape variants were neutralized during the course of infection, all 22 longitudinal Envs were assayed for neutralization with 16-month plasma (**F**); it was from PBMC collected at this time point that the two autologous R880F mAbs, 19.3H-L1 and 19.3H-L3, were derived. Percent viral infectivity, as adjusted against wells containing no test plasma, is depicted on the vertical axis; reciprocal plasma dilutions are plotted along the horizontal axis in a logarithmic fashion. Each curve represents a single Env-plasma combination, and error bars demonstrate the standard error of the mean of two independent experiments using duplicate wells (0-month Envs = circles, 2-month Envs = triangles, 5-month Envs = inverted triangles, 7-month Envs = squares, 10-month Envs = diamonds). Colored lines (2-A9/2-A13 in magenta, 2-B31 in red, 2-B12 in cyan, and 5-B52 in green) indicate Envs that succumbed to neutralization, in varying combinations, by the isolated R880F mAbs.

Each Env-pseudotyped virus was assayed against autologous plasma contemporaneous to its date of isolation in the Tzm-bl neutralization assay. Plasma samples from 2-, 5-, 7-, and 10-months, but not 0-months, potently neutralized the founder Envs 0-A6 and 0-B24 (Figure 1). All longitudinal Envs were at least one log less sensitive to neutralization by contemporaneous plasma than the founder Envs and were, therefore, considered humoral escape variants (Figure 1B–E). These 0-, 2-, 5-, 7-, and 10-month Envs all succumbed to neutralization by plasma collected at 16-months post-seroconversion (Figure 1F). Hence, the induction of *de novo* nAbs against contemporaneous escape variants, which we and others have previously described [26]–[30], also occurred during the first year and a half of infection in R880F. In this subtype A HIV-1 infected subject, a potent nAb response was detected by 2-months following the first antibody positive time point and initiated repeated rounds of neutralization and viral escape.

### Residues responsible for early nAb escape coalesce to a potential V3-proximal epitope with alpha2 helix participation

**Figure 2:**
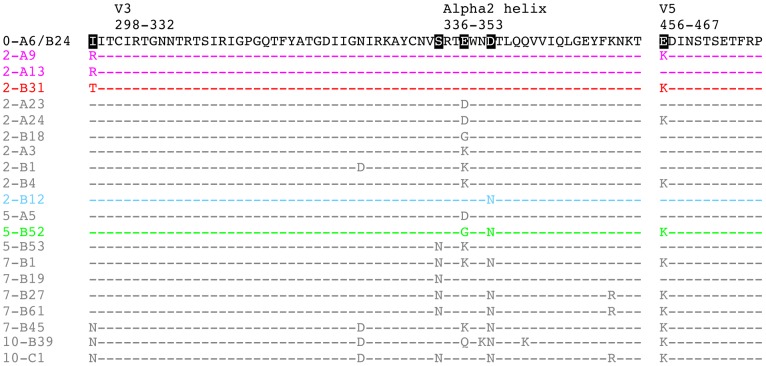
Amino acid alignment of longitudinal R880F Envs. Longitudinal Env amino acid sequences from 20 R880F contemporaneous plasma escape variants were aligned and examined using Sequencher v5.0 and Geneious v5.0.3 software, with particular emphasis on the three mutational hot spots–C2, the alpha2 helix in C3, and V5–that developed during early infection, as compared to the transmitted/founder Env (0-A6/B24). Dashes represent conserved positions. Subject-specific amino acid numbering labels the demarcated regions at their points of origination. Five residues including 295, 335, 338, 341, and 456 (HXB2 residues 293, 334, 337, 340, and 460) were specifically interrogated to define their contributions to nAb evasion and have been highlighted in black in 0-A6/B24 for easy identification. Colored sequences (2-A9/2-A13 in magenta, 2-B31 in red, 2-B12 in cyan, and 5-B52 in green) indicate Envs that succumbed to neutralization, in varying combinations, by the isolated R880F mAbs. Note that additional differences from the transmitted/founder Env, which occurred over time in these sequences but are not diagrammed here, may be viewed in Figure S1.

To localize the earliest nAb target and elucidate consequent mechanisms of viral escape, full-length amino acid Env sequences for all 2-month nAb escape variants shown in Figure 1B were aligned and inspected for the presence of mutational hot spots. Amino acid changes concentrated in three regions of gp120 at 2-months: in C2 immediately preceding the beginning of V3, in the alpha2 helix in C3, and in V5. Figure 2 specifically diagrams these segments of gp120; Figure S1 includes the full gp160 alignment of all 22 R880F Envs. The isoleucine at position 295 (I295; HXB2 residue 293) in C2 mutated to arginine (I295R) in two Envs or threonine (I295T) in one Env (Figure 2). Additionally, glutamic acid E338 in the alpha2 helix (HXB2 residue 337) became three different residues including aspartic acid (E338D), glycine (E338G), and lysine (E338K) in six Envs (Figure 2). Of note, compared to the founder Env sequence, E338K was the sole mutation in the entire 2-A3 Env sequence (Figure S1). We concluded, then, that this single mutation directly mediated nAb escape. The aspartic acid at position 341 (D341; HXB2 residue 340), also in the alpha2 helix, changed to asparagine (D341N) in one Env (Figure 2). Finally, the glutamic acid at position 456 (E456; HXB2 residue 460) in V5 switched to lysine (E456K) in four Envs (Figure 2).

**Figure 3:**
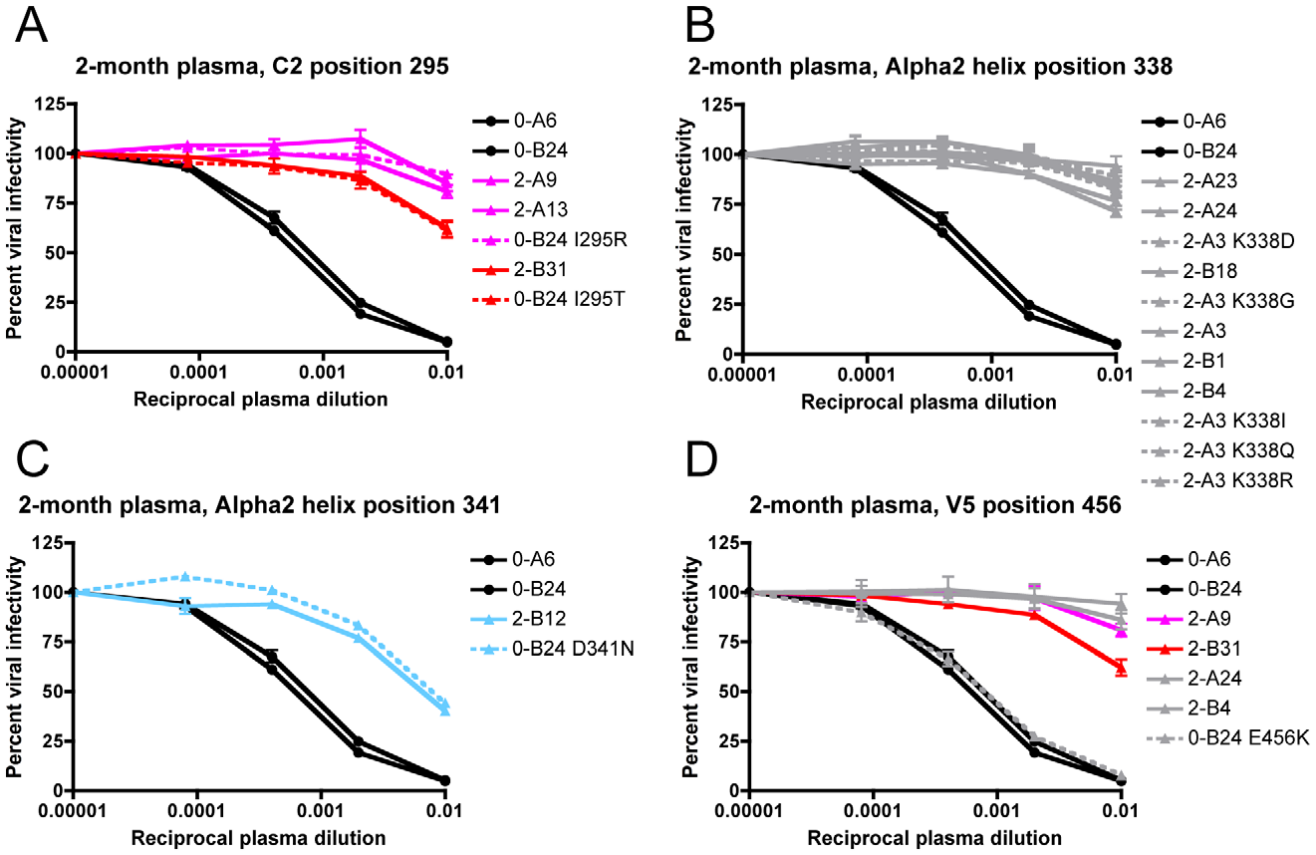
Determination of the earliest R880F nAb escape signatures in Env. Suspected 2-month nAb escape mutations from four different amino acid positions were introduced by site-directed mutagenesis into the transmitted/founder Env 0-B24 or escape Env 2-A3, which differed from the founder only at the site of mutation introduction. The transmitted/founder and wild-type 2-month Envs (solid lines) along with site-directed mutant Envs (dashed lines) were pseudotyped and assayed with 2-month plasma to determine if substitutions at C2 295 (**A**), alpha2 helix 338 (**B**), alpha2 helix 341 (**C**), or V5 456 (**D**) (HXB2 residues 293, 337, 340, or 460) could individually confer resistance. Percent viral infectivity, as adjusted against wells containing no test plasma, is depicted on the vertical axis; reciprocal plasma dilutions are plotted along the horizontal axis in a logarithmic fashion. Each curve represents a single Env-plasma combination, and error bars demonstrate the standard error of the mean of two independent experiments using duplicate wells (0-month Envs = circles, 2-month Envs and representative point mutants = triangles). Colored lines (2-A9/2-A13/0-B24 I295R in magenta, 2-B31/0-B24 I295T in red, and 2-B12/0-B24 D341N in cyan) indicate Envs that succumbed to neutralization, in varying combinations, by the isolated R880F mAbs.

The potential escape mutations at I295, D341, and E456 were introduced into the founder Env 0-B24 by site-directed mutagenesis to determine if these alterations could individually switch the founder Env phenotype from sensitive to resistant when assayed for neutralization by 2-month plasma. In addition, amino acid changes were introduced into escape Env 2-A3 at K338 to determine whether these mutants maintained nAb resistance. The I295R and I295T substitutions in C2 independently conferred nAb escape, with I295R producing a slightly higher level of resistance that was most evident at the 1∶100 dilution of plasma (Figure 3A). For position 338, two naturally occurring substitutions (E338D from 2-A23/2-A24 and E338G from 2-B18, see Figure 2) and three artificially introduced mutations (K338I, K338Q, and K338R) independently reproduced escape Env 2-A3's wild-type level of resistance, arguing that any change at this position could provide full escape from neutralization (Figure 3B). Thus, the degree of neutralization resistance conferred by changes at I295, but not at E338, varied by the amino acid substitution. Introduction of D341N into the alpha2 helix of the founder Env 0-B24 also recapitulated the wild-type resistance level of 2-B12 (Figure 3C). Because the I295, E338, and D341 escape mutations occurred independently in the 2-month Envs, each represents a distinct lineage for escape (Figure 2). In addition, the potency of resistance was substitution-specific; I295R/T and E338D/G/K produced the highest levels of resistance, while D341N lagged somewhat behind and provided partial resistance. In contrast, the E456K mutation in V5 exerted no overt influence on neutralization phenotype when introduced into the founder Env 0-B24, despite being carried in nearly half of the 2-month escape Envs (Figure 3D). Overall, at 2-months, the viral population utilized a common amino acid substitution mechanism that diverged down three discrete escape pathways, each of which conferred nAb resistance.

**Figure 4:**
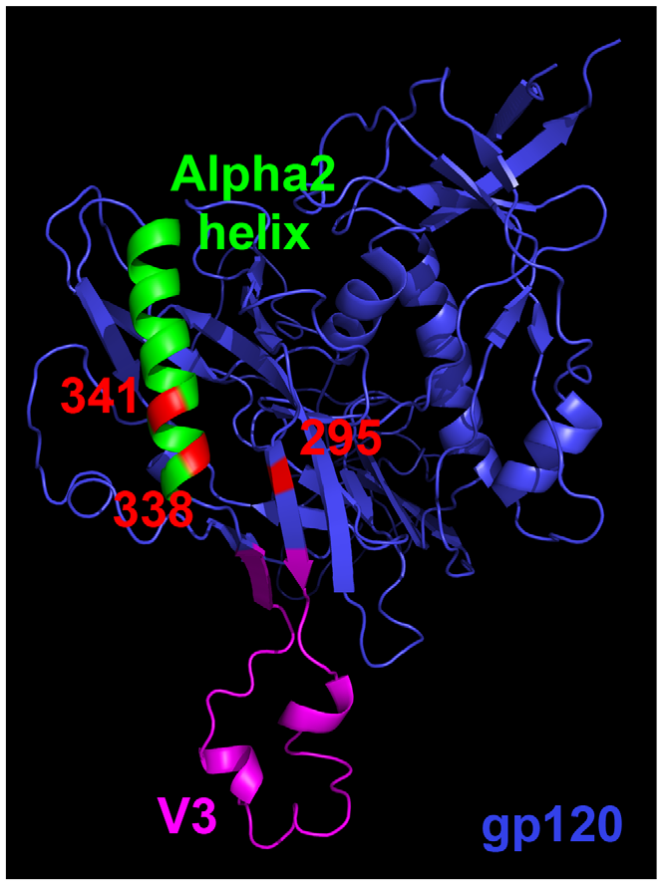
Homology model of R880F gp120. A 3-dimensional gp120 monomer (blue) based on the R880F transmitted/founder Env 0-A6/B24 sequence was homology modeled from existing gp120 structures (see Materials and Methods) and spatially oriented using MacPyMOL software to illuminate the region targeted by the earliest nAbs in this subject. Functional domains such as the alpha2 helix (green) and V3 (magenta) are delineated, and subject-specific amino acid numbering indicates positions that mutated at 2-months post-seroconversion to confer nAb escape. These residues (295, 338, and 341 in red) nest together in a putative epitope.

Though positions 295, 338, and 341 appear disparate in the linear gp120 sequence, these residues cluster when plotted onto a 3-dimensional representation of the R880F founder Env sequence, which was modeled using all existing structures for CD4-bound HIV-1 gp120 (Figure 4). This proposed epitope emerges near the base of the V3 domain, which is well exposed on the native trimer and is also targeted by the broadly neutralizing, glycan-dependent mAb PGT128 [8]. The spatial proximity of these three residues provides evidence for a single nAb epitope during early subtype A HIV-1 infection and an explanation for why a substitution at any one of the three positions independently caused nAb resistance.

### Autologous mAbs neutralize initial escape variants and typify a subsequent wave of humoral pressure

**Figure 5:**
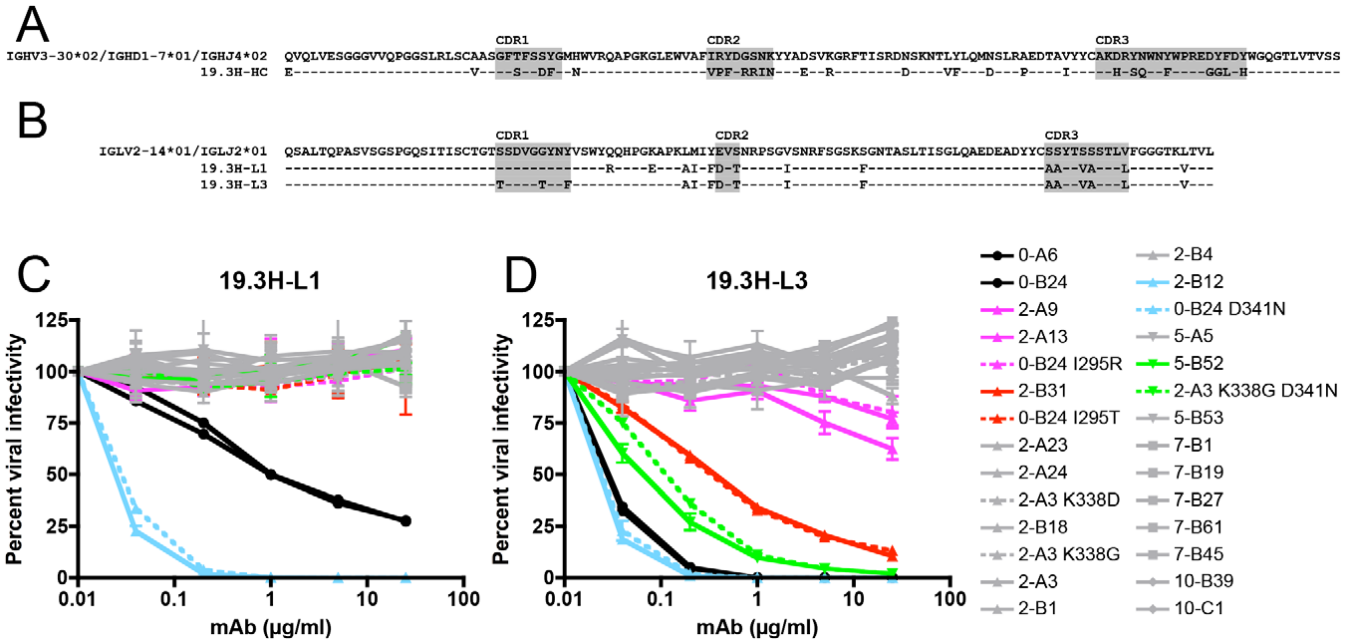
Amino acid alignment of R880F immunoglobulin heavy and light chain variable domains and neutralization by R880F mAbs 19.3H-L1 and 19.3H-L3. Germline heavy and light chain gene segment utilization was determined by SoDA, a somatic diversification analysis program [32], and amino acid sequences were aligned and examined using Sequencher v5.0 and Geneious v5.0.3 software. Dashes represent conserved positions. Complementarity-determining regions (CDRs) are highlighted in gray. The two R880F mAbs share a common heavy chain, 19.3H-HC (**A**), which utilizes V3-30*02, D1-7*01, and J4*02 gene families, while the somatically related light chains 19.3H-L1 and 19.3H-L3 (**B**) employ V2-14*01 and J2*01 gene families and differ from each other at five positions in and just downstream of CDR1. Heavy chain 19.3H-HC, when paired with either 19.3H-L1 (**C**) or 19.3H-L3 (**D**), was evaluated for neutralization against pseudotyped R880F wild-type (solid lines) and site-directed mutant Envs (dashed lines). Percent viral infectivity, as adjusted against wells containing no mAb, is depicted on the vertical axis; mAb concentrations (in µg/ml) are plotted along the horizontal axis in a logarithmic fashion. Each curve represents a single Env-mAb combination, and error bars demonstrate the standard error of the mean of two independent experiments using duplicate wells (0-month Envs = circles, 2-month Envs and representative point mutants = triangles, 5-month Envs and a representative point mutant = inverted triangles, 7-month Envs = squares, 10-month Envs = diamonds). Colored lines (2-A9/2-A13/0-B24 I295R in magenta, 2-B31/0-B24 I295T in red, 2-B12/0-B24 D341N in cyan, and 5-B52/2-A3 K338G D341N in green) indicate Envs that succumbed to neutralization, in varying combinations, by the isolated R880F mAbs.

During HIV-1 infection, the antibodies circulating in patient plasma could ostensibly represent a heterogeneous pool with varying epitope specificities. Although we were able to identify a single, early nAb target in subject R880F using autologous plasma and 3-dimensional modeling, this epitope could be recognized by a polyclonal nAb response mediated by more than one B cell [31]. To illuminate the characteristics of individual monoclonal effectors, we PCR amplified and cloned antibody VH and VL genes from memory B cells present in a cryopreserved R880F peripheral blood mononuclear cell (PBMC) sample collected at 16-months post-seroconversion (Table 1). Multiple VHs and VLs were obtained, but only one VH, named 19.3H-HC, neutralized the founder Env when combined with either of two highly related VLs. Sequence analysis revealed that the R880F VH utilized IGHV3-30*02, IGHD1-7*01, and IGHJ4*02 gene segments based on matching within the SoDA database [32] and demonstrated 23.2% mutation across its framework (FWR) and complementarity-determining regions (CDR), as compared with germline at the amino acid level (Figure 5A). The VLs, named 19.3H-L1 and 19.3H-L3, were clonal relatives, both using IGLV2-14*01 and IGLJ2*01 gene segments based on matching within the SoDA database [32] and exhibiting mutation rates of 13.6% and 14.5% from the putative germline, respectively (Figure 5B). Five total amino acid differences between the 19.3H-L1 and 19.3H-L3 VLs congregated in and around CDR1: 19.3H-L3 contained two threonines (T) and one phenylalanine (F) in CDR1 that were not present in 19.3H-L1, while arginine (R) and glutamic acid (E) residues arose just downstream of CDR1 in the FWR2 region of 19.3H-L1 that were not present in 19.3H-L3 (Figure 5B). The VL CDR3 domains of 19.3H-L1 and 19.3H-L3 were identical and contained five amino acid differences from the putative germline. The two R880F mAbs produced by combination of 19.3H-HC and 19.3H-L1 or 19.3H-L3 are hereafter referenced solely by their VL designations.

Figure 5 demonstrates that both 19.3H-L1 (C) and 19.3H-L3 (D) neutralized the founder Envs 0-A6 and 0-B24, although 19.3H-L3 did so with approximately one log greater potency. In addition to neutralizing the founder Env, both mAbs neutralized the 2-month plasma escape Env 2-B12 with similar potencies. 19.3H-L3 also neutralized plasma escape variants 5-B52 and 2-B31 potently, and 2-A9 and 2-A13 to a much lesser extent. The remaining 2- and 5-month escape variants, and all 7- and 10-month escape variants were resistant to both mAbs. This result suggests that the mAbs are representative of those that circulated within the first few (2–5) months of infection; because they were isolated from memory B cells, 19.3H-L1 and 19.3H-L3 do not reflect the ability of the 16-month plasma nAbs to neutralize all longitudinal R880F Envs (Figure 1F, Table 2). To provide evidence for the specificity and authenticity of 19.3H-L1 and 19.3H-L3, the common VH, 19.3H-HC, was co-transfected with other autologous VL genes from two randomly selected R880F B cell wells. One VL utilized the same IGLV2-14*01 gene segment as 19.3H-L1 and 19.3H-L3 (Figure S2A,E); one did not (Figure S2B,E). Conversely, the 19.3H-L3 VL was paired with an autologous VH from a different R880F B cell well (Figure S2C,E). All three chimeric antibody supernatants were assayed for activity against a smaller panel of ten longitudinal R880F Envs, and no neutralizing activity was observed (Figure S2A–C), suggesting that stochastic pairing of R880F VHs and VLs does not confer neutralizing activity.

**Table 2:** IC_50_ values for autologous plasma/mAbs with R880F wild-type and mutant Envs.

Envelope^a^	Contemp.^b^ plasma	16-month plasma	19.3H-L1^d^	19.3H-L3^d^	Description	295^f^	335^f^	338^f^	341^f^
0-A6	<100	5157	1.794	0.095	Founder	I	S	E	D
0-B24	<100	5763	1.519	0.094	Founder	I	S	E	D
0-A6 I295N	nd^c^	nd	>25	>25	Mutant	N*	S	E	D
0-B24 I295R	nd	nd	>25	>25^e^	Mutant	R	S	E	D
0-B24 I295T	nd	nd	>25	0.515	Mutant	T	S	E	D
0-A6 S335N	nd	nd	>25	>25	Mutant	I	N**	E	D
0-B24 D341N	nd	nd	0.095	0.092	Mutant	I	S	E	N
2-A3	<100	1447	>25	>25	2 m Escape	I	S	K	D
2-A3 K338D	nd	nd	>25	>25	Mutant	I	S	D	D
2-A3 K338G	nd	nd	>25	>25	Mutant	I	S	G	D
2-A3 K338I	nd	nd	>25	>25	Mutant	I	S	I	D
2-A3 K338Q	nd	nd	>25	>25	Mutant	I	S	Q	D
2-A3 K338R	nd	nd	>25	>25	Mutant	I	S	R	D
2-A3 K338G D341N	nd	nd	>25	0.207	Mutant	I	S	G	N
2-A9	<100	4459	>25	>25^e^	2 m Escape	R	S	E	D
2-A13	<100	3221	>25	>25^e^	2 m Escape	R	S	E	D
2-A23	<100	4779	>25	>25	2 m Escape	I	S	D	D
2-A24	<100	5279	>25	>25	2 m Escape	I	S	D	D
2-B1	<100	2491	>25	>25	2 m Escape	I	S	K	D
2-B4	<100	2387	>25	>25	2 m Escape	I	S	K	D
2-B12	126	3516	0.092	0.092	2 m Escape	I	S	E	N
2-B12 I295N	nd	nd	>25	>25	Mutant	N*	S	E	N
2-B12 S335N	nd	nd	>25	>25	Mutant	I	N**	E	N
2-B12 S335Q	nd	nd	1.981	0.113	Mutant	I	Q	E	N
2-B18	<100	3336	>25	>25	2 m Escape	I	S	G	D
2-B31	<100	4594	>25	0.512	2 m Escape	T	S	E	D
5-A5	<100	1891	>25	>25	5 m Escape	I	S	D	D
5-B52	<100	1184	>25	0.152	5 m Escape	I	S	G	N
5-B52 S335N	nd	nd	>25	>25	Mutant	I	N**	G	N
5-B53	<100	468	>25	>25	5 m Escape	I	N**	K	D
7-B1	<100	558	>25	>25	7 m Escape	I	N**	K	N
7-B19	<100	106	>25	>25	7 m Escape	I	N**	E	D
7-B27	<100	462	>25	>25	7 m Escape	I	N**	E	N
7-B45	<100	539	>25	>25	7 m Escape	N*	S	K	N
7-B61	<100	539	>25	>25	7 m Escape	I	N**	E	N
10-B39	<100	990	>25	>25	10 m Escape	N*	S	Q	N
10-C1	<100	998	>25	>25	10 m Escape	N*	N**	E	N

a Longitudinal wild-type and mutant Envs were assayed for neutralization sensitivity to autologous plasma and mAbs 19.3H-L1 and 19.3H-L3 in Tzm-bl cells. Average IC_50_ values for two independent experiments using duplicate wells are given in the second through fifth columns.

b Autologous plasma samples assayed in the second column were contemporaneous with envelope isolation dates (e.g. 0-month plasma with 0-month Envs, 2-month plasma with 2-month Envs, etc.).

c nd, not done.

d 19.3H-L1 and 19.3H-L3 were isolated from a 16-month cryopreserved R880F PBMC sample.

e These envelopes, though technically demonstrating IC_50_ values greater than 25 µg/ml, saw slight neutralization at the highest concentration of antibody tested.

f The four right-most columns detail which amino acids appear at the specified Env residues (295, 335, 338, and 341). N* denotes the introduction of a potential N-linked glycosylation site, while N** marks where such a site has been shifted downstream from a previously existing site in the Env sequence.

To map the specificity of mAbs 19.3H-L1 and 19.3H-L3 in finer detail, we utilized the point mutants from Figure 3, with the addition of double mutant 2-A3 K338G D341N, which was representative of escape Env 5-B52. As previously mentioned, 19.3H-L1 neutralized Env 2-B12 in addition to the founder Envs; Env 2-B12 was the only Env in the panel that shared with the founder Env all three unmutated residues at positions I295, S335, and E338 (Figure 2, Table 2). A change at any one of these positions resulted in resistance to 19.3H-L1 neutralization (Figure 5C, Table 2). 19.3H-L1 neutralized 2-B12 more potently than the founder Envs; this was directly attributed to D341N, as this single substitution introduced into Env 0-B24 (0-B24 D341N) increased the founder Env's sensitivity to that of 2-B12 (Figure 5C, Table 2). Despite sharing a common VH with 19.3H-L1, 19.3H-L3 demonstrated a distinct pattern of specificity. In contrast to 19.3H-L1, 19.3H-L3 neutralized the founder and 2-B12 Envs equivalently. In this case, then, the D341N mutation (0-B24 D341N) had very little effect on the neutralization phenotype (Figure 5D). 19.3H-L3 also neutralized Envs carrying the I295T substitution (0-B24 I295T and 2-B31) but displayed a much weaker level of neutralization capacity against Envs containing the I295R substitution (0-B24 I295R, 2-A9, and 2-A13). 19.3H-L3 neutralized Envs containing the E338G substitution when it occurred in the presence of D341N (5-B52 and 2-A3 K338G D341N) but not when E338G (or any other E338 substitution) occurred in isolation (Figure 5D, Table 2). R880F mAb 19.3H-L3, therefore, had potent neutralizing activity against two Envs (5-B52 and 2-B31) and modest activity against two Envs (2-A9 and 2-A13) that were resistant to contemporaneous plasma and to mAb 19.3H-L1. Hence, the mutational program at positions 295, 338, and 341, first witnessed at 2-months post-seroconversion to facilitate immune evasion (Figure 3), likely fueled subsequent rounds of nAb recognition, and mutations that originally evolved the virus toward an escaped phenotype here conferred sensitivity to somatically related autologous mAbs (Figure 5, Table 2).

**Figure 6:**
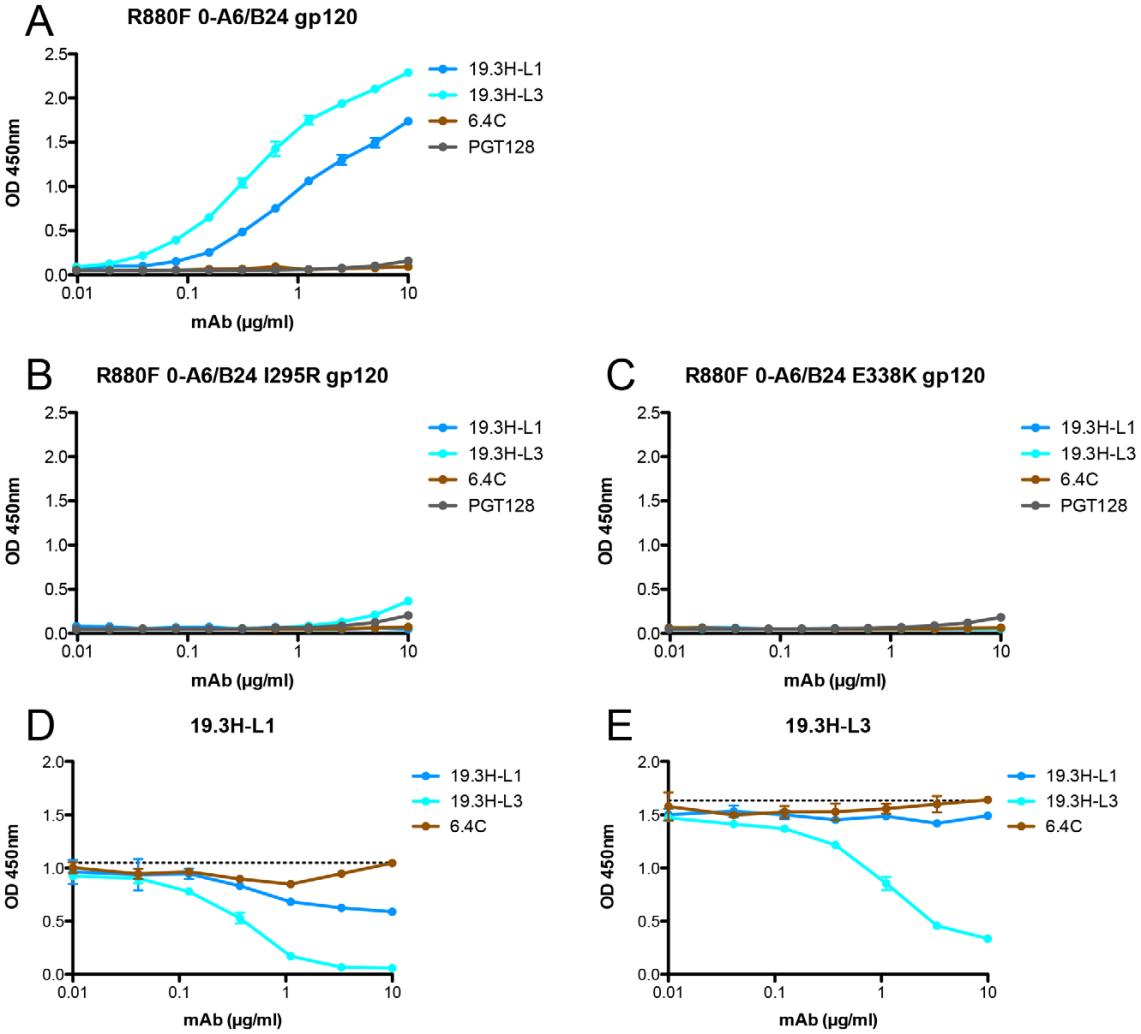
gp120 binding by and competition of R880F mAbs 19.3H-L1 and 19.3H-L3. The baseline binding of four biotinylated mAbs, 19.3H-L1, 19.3H-L3, 6.4C, or PGT128, was evaluated by ELISA with three R880F gp120 proteins: (**A**) wild-type 0-A6/B24, (**B**) point mutant 0-A6/B24 I295R, and (**C**) point mutant 0-A6/B24 E338K. R880F mAbs 19.3H-L1 and 19.3H-L3 were then competed with themselves, each other, and the negative control antibody, 6.4C. For the competition ELISAs, plates were coated with wild-type R880F 0-A6/B24 gp120 protein, pre-incubated with serially-diluted 19.3H-L1, 19.3H-L3, or 6.4C, washed, and then incubated with 1 µg/ml biotinylated 19.3H-L1 (**D**) or 19.3H-L3 (**E**). From data in (**A**), 1 µg/ml was selected as a point of non-saturated binding. The horizontal dashed lines in (**D**) and (**E**) represent 100% binding of biotinylated 19.3H-L1 or 19.3H-L3, at 1 µg/ml in the absence of competitor, respectively. Optical density values at 450 nm are depicted on the vertical axis; mAb concentrations (in µg/ml) are plotted along the horizontal axis in a logarithmic fashion. Error bars demonstrate the standard error of the mean of two independent experiments.

To ascertain if 19.3H-L1 and 19.3H-L3 would compete for Env binding, three R880F gp120 monomeric proteins (the 0-A6/B24 founder Env gp120, and mutants containing I295R or E338K) were synthesized, purified, and employed in a competition ELISA assay. To first establish a baseline level of binding, the R880F mAbs were biotinylated and incubated with wild-type 0-A6/B24 gp120 protein. 19.3H-L3 demonstrated more robust binding, as compared to 19.3H-L1; the negative control mAb 6.4C (directed against a highly specific epitope in V1V2 [31]), and the broadly neutralizing mAb PGT128 [8], which shares epitope space with the R880F mAbs, both failed to bind (Figure 6A). Consistent with the neutralization data in Figure 5, neither R880F mAb could bind detectably to the I295R or E338K mutant gp120 proteins (Figure 6B–C). Wild-type 0-A6/B24 gp120 protein was then pre-incubated with 19.3H-L1, 19.3H-L3, or the negative control antibody 6.4C, washed, and incubated with either biotinylated 19.3H-L1 (Figure 6D) or 19.3H-L3 (Figure 6E) to discern if initial pre-incubation could block secondary binding. 19.3H-L1 modestly competed with itself (Figure 6D) but could not effectively compete for binding with 19.3H-L3 (Figure 6E). Conversely, 19.3H-L3 strongly competed with both itself (Figure 6E) and 19.3H-L1 (Figure 6D). Thus, 19.3H-L3 neutralizes a greater number of R880F Envs than 19.3H-L1, binds more strongly to the founder 0-A6/B24 gp120, and neutralizes the Env 0-A6/B24 pseudovirus more potently, underscoring the significance of VL alterations where antigen recognition and neutralization efficacy are concerned.

### Crystal structures reveal the neutral, planar epitope contact surfaces and explain the antigen- binding properties of mAbs 19.3H-L1 and 19.3H-L3

**Figure 7:**
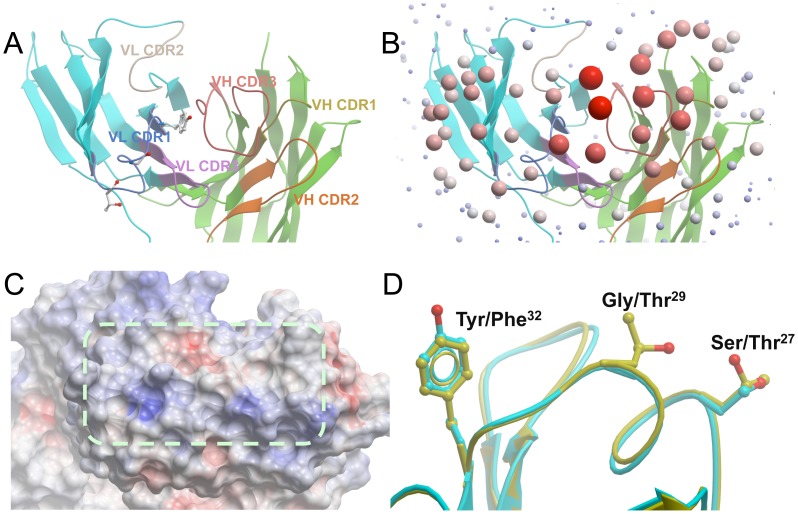
Crystal structures of R880F mAbs 19.3H-L1 and 19.3H-L3. (**A**) CDR loops. A top view looking down at the antigen-binding site of 19.3H-L1 represented by ribbons. The framework regions of the light chain and heavy chain are colored cyan and green, respectively, while each CDR loop is colored separately. The side chains of the three VL CDR1 residues different between 19.3H-L1 and 19.3H-L3 are displayed. (**B**) ODA analysis of the Fab 19.3H-L1. The size/redness of each sphere is proportional to the binding strength of the region indicated. Note that the antigen-binding site is centered at VL CDR1 and VH CDR3. (**C**) The electrostatic surface potentials of the antigen-binding site of 19.3H-L1. Red and blue coloration represents the negatively and positively charged regions, respectively, while a dashed line encircles the flat surface of the antigen-binding site. (**D**) The three VL CDR1 amino acid differences, S27T, G29T, and Y32F, between 19.3H-L1 (cyan) and 19.3H-L3 (yellow).

To interrogate the antigen-binding site characteristics of R880F mAbs that influenced their distinct neutralization profiles, crystal structures of the 19.3H-L1 and 19.3H-L3 Fabs were determined to the resolutions of 1.7 Å (Figure 7A) and 2.7 Å, respectively (Table S1). Although the two Fabs were crystallized in different space groups, the resultant structures were highly similar, with root mean square deviations less than 1 Å when all of the Cα atoms were superimposed (data not shown). Several structural analyses were employed, including calculations of Optical Docking Area (ODA, shown in Figure 7B, which predicted the antigen-binding sites by calculating the desolvation free energy of the surfaces), surface pockets, and electrostatic surface potentials. ODA analyses indicated that the antigen-binding sites of 19.3H-L1 and 19.3H-L3 were very flat, forming roughly rectangular shapes approximately 15 Å wide and 30 Å long on top of the six CDR loops (Figure 7C). No pockets existed in these binding surfaces, and the shared CDR H3, although it was 18 amino acids long (Kabat numbering scheme [33]), did not protrude. Such flat antigen-binding sites likely interact with epitopes formed by residues also on planar surfaces (i.e. flat-surface antigen-antibody contacts). Electrostatic surface potential analyses showed that the 19.3H-L1 and 19.3H-L3 antigen-binding sites were essentially neutral; a couple of slightly positive regions along one side of the rectangular contact area counterbalanced a slightly negative opposite region (Figure 7C, blue and red patches, respectively).

Three CDR1 residues that differed between 19.3H-L1 and 19.3H-L3 (Ser/Thr at residue 27, Gly/Thr at residue 29, and Tyr/Phe at residue 32; Kabat numbering scheme [33]; Figure 7D) did not create any substantial structural differences between the two antigen-binding sites. These changes did, however, have the potential to influence antigen-antibody interactions. The Tyr in 19.3H-L1 to Phe in 19.3H-L3 change at residue 32 likely increased the hydrophobicity at the center of the antigen-binding site, which may have augmented hydrophobic interactions with the antigen. The Gly to Thr mutation at residue 29 added a polar side chain with additional hydrogen binding possibilities. Finally, the Ser to Thr substitution at residue 27 provided a more stable side chain. As a group, these VL alterations probably enhanced the antigen-binding affinity of 19.3H-L3, explaining its increased autologous neutralization breadth.

### Addition and/or shifting of potential N-linked glycosylation sites mediate escape from mAbs 19.3H-L1 and 19.3H-L3

**Figure 8:**
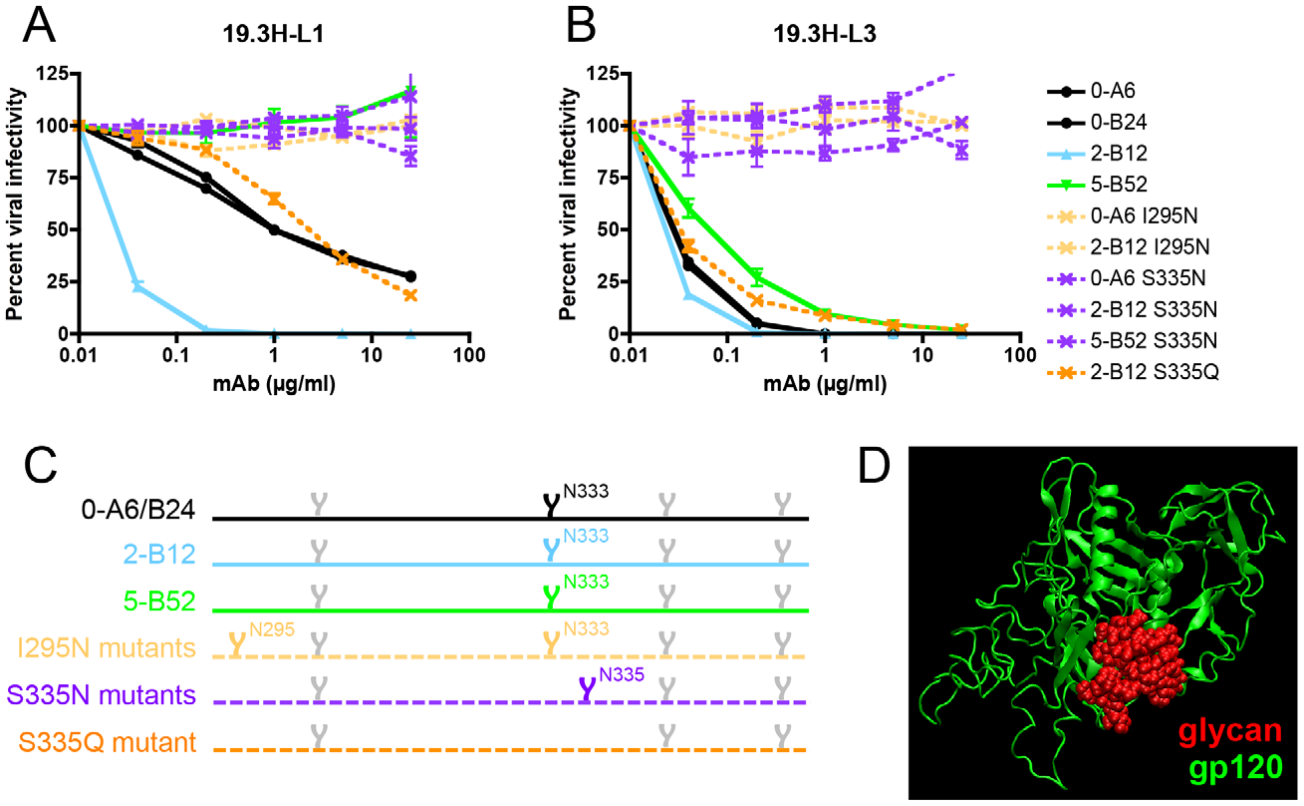
Escape from mAbs 19.3H-L1 and 19.3H-L3 by glycan addition and/or shifting. To investigate how longitudinal viruses, namely 7- and 10-month Envs, could harbor the humoral vulnerability associated with mutation D341N whilst maintaining neutralization resistant phenotypes, two potential compensatory mutations were investigated. I295N, which inserts a PNGS near the N-terminus of V3, was introduced by site-directed mutagenesis into two mAb-sensitive Envs, 0-A6 and 2-B12 (light orange). S335N, which shifts a PNGS closer to the N-terminus of the alpha2 helix, was similarly created in 0-A6, 2-B12, and 5-B52 (purple). Wild-type (solid lines) and site-directed mutant Envs (dashed lines) were pseudotyped and assayed with mAbs 19.3H-L1 (**A**) and 19.3H-L3 (**B**). To determine if mAb resistance was glycan-dependent, an S335Q substitution that destroyed the N333 PNGS was also created in Env 2-B12 (dark orange). Percent viral infectivity, as adjusted against wells containing no mAb, is depicted on the vertical axis; mAb concentrations (in µg/ml) are plotted along the horizontal axis in a logarithmic fashion. Each curve represents a single Env-mAb combination, and error bars demonstrate the standard error of the mean of two independent experiments using duplicate wells. In (**C**), the V3/alpha2 helix portion of the labeled Envs has been conceptualized with glycan forks, each of which represents a PNGS in the corresponding amino acid sequences. Glycans of particular interest (N295, N333, and N335) are designated using R880F-specific numbering. In (**D**), the proposed escape glycans N295 and N335 (red) have been modeled onto the R880F 0-B24 Env gp120 monomer (green) to illustrate how such masking could obscure underlying epitopes and prevent recognition by mAbs 19.3H-L1 and 19.3H-L3.

As demonstrated in Figure 5, D341N appeared to be detrimental to the preservation of a neutralization-resistant phenotype, in the context of mAbs 19.3H-L1 and 19.3H-L3 during early infection. This mutation was, nonetheless, retained in later escape Envs. Inspection of the 7- and 10-month Env sequences containing D341N revealed that they had acquired additional substitutions, I295N (HXB2 residue 293) and/or S335N (HXB2 residue 334), absent from earlier Envs (Figure 2); each of these mutations affected a potential N-linked glycosylation site (PNGS). Accordingly, we hypothesized that these co-traveling mutations compensated for the vulnerability associated with D341N in a PNGS-dependent manner. To explore this, the I295N substitution, which created a PNGS, was introduced into two mAb-sensitive Envs: 0-A6 and 2-B12. The I295N versions of these two Envs displayed high-level resistance against mAbs 19.3H-L1 and 19.3H-L3 (Figure 8A–B, Table 2). Similarly, the S335N substitution, which also incorporated a PNGS, was inserted in three mAb-sensitive Envs: 0-A6, 2-B12, and 5-B52. The S335N versions of these three Envs also became highly resistant to 19.3H-L1 and 19.3H-L3 (Figure 8A–B, Table 2). The S335N substitution shifted a well-conserved PNGS sequon at position 333 (HXB2 residue 332; Figure 8C) that is targeted by broadly neutralizing mAbs PGT128 and 2G12 [8], [34], [35]. To determine if the observed mAb resistance was glycan-dependent, an S335Q substitution was created in Env 2-B12. Unlike S335N, which shifted the N333 sequon down two positions, S335Q destroyed the N333 sequon altogether (Figure 8C). The resulting mutant, 2-B12 S335Q, was two logs less sensitive to neutralization by mAb 19.3H-L1 than the parental Env 2-B12, but did not reach the high level of resistance achieved by 2-B12 S335N; in contrast, S335Q had only a slight effect on neutralization by mAb 19.3H-L3 (Figure 8A–B, Table 2). High-level resistance against mAbs 19.3H-L1 and 19.3H-L3, therefore, required the addition and/or shifting of PNGS sequons, but amino acid substitution S335Q also provided partial resistance that was much more effective against mAb 19.3H-L1. Together, the data strongly support a mechanism of mAb escape that was PNGS-dependent and may have introduced glycans capable of obscuring the V3-proximal space recognized by 19.3H-L1 and 19.3H-L3 (Figure 8D). Nevertheless, the two mAbs–common heavy chain notwithstanding–appear to recognize subtly distinct epitopes.

### R880F exhibits modest heterologous neutralization breadth by 16-months post-infection

**Figure 9:**
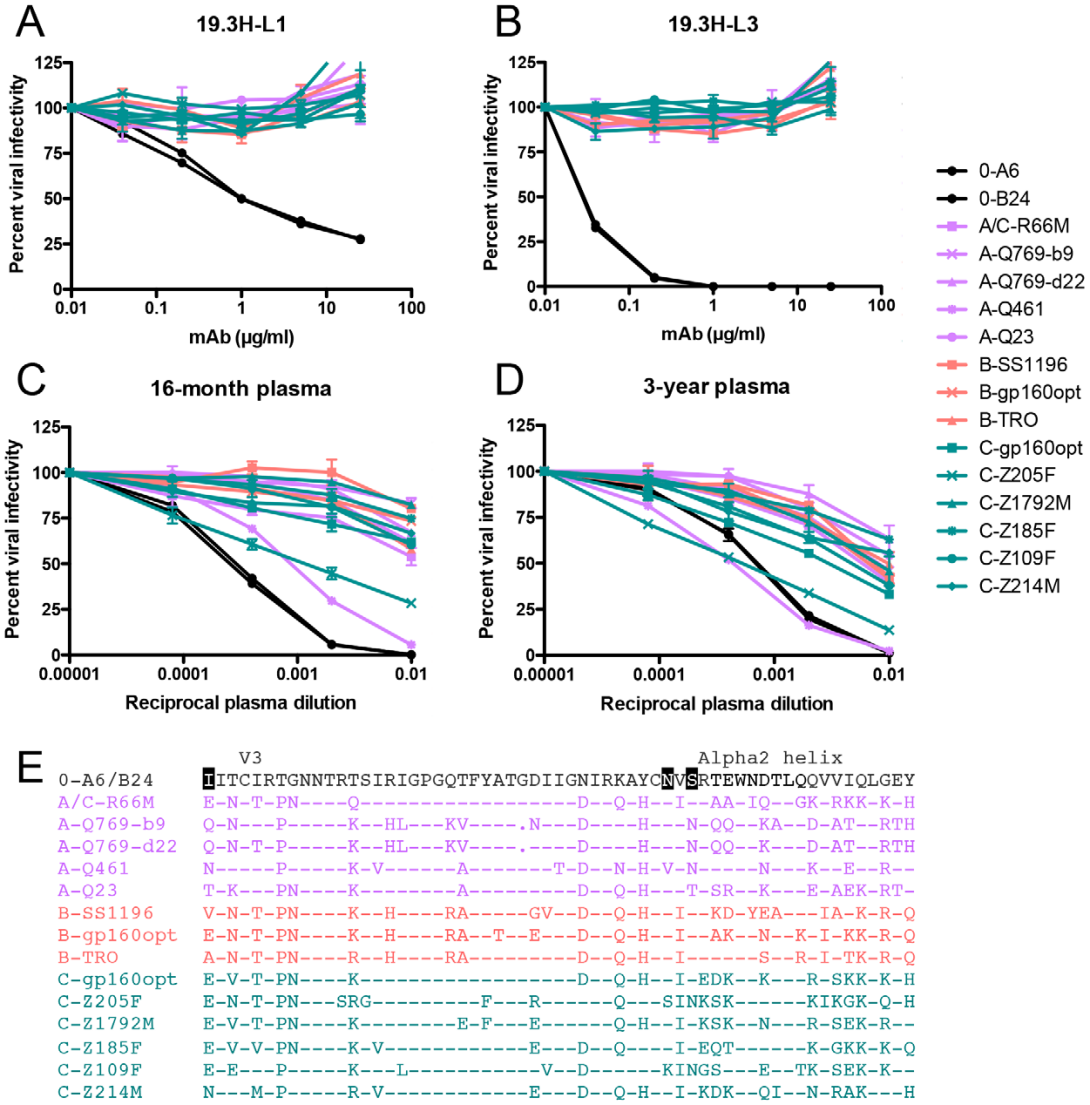
Heterologous neutralization breadth in R880F. The two R880F mAbs, 19.3H-L1 (**A**) and 19.3H-L3 (**B**), in conjunction with 16-month (**C**) and 3-year (**D**) autologous plasma were evaluated for cross-neutralizing capacity against virions pseudotyped with fourteen heterologous HIV-1 Envs from three clades (A/C recombinant and subtype A Envs = lavender, subtype B Envs = coral, subtype C Envs = teal). Two 0-month Envs (0-A6/B24) representative of the transmitted/founder sequence are included in each of these panels. Percent viral infectivity, as adjusted against wells containing no mAb or test plasma, is depicted on the vertical axis; mAb concentrations (in µg/ml) or reciprocal plasma dilutions are plotted along the horizontal axis in a logarithmic fashion. Each curve represents a single Env-mAb or Env-plasma combination, and error bars demonstrate the standard error of the mean of two independent experiments using duplicate wells. V3/alpha2 helix amino acid sequences were aligned and examined using Sequencher v5.0 and Geneious v5.0.3 software (**E**). Dashes represent conserved positions; dots represent gaps. Significant PNGS sequons (N295, N333, N335) are highlighted in black at their points of origin.

**Figure 10:**
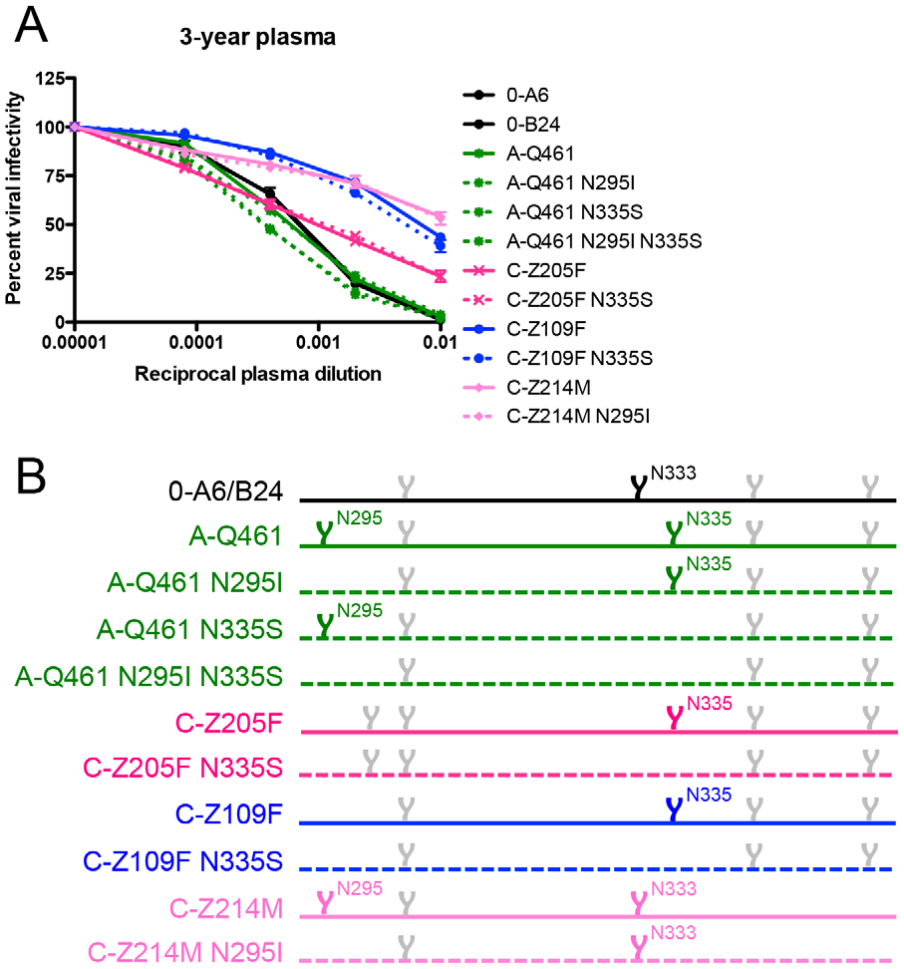
Contribution of specific glycans to heterologous neutralization breadth in R880F. To gauge if nAb responses continued to exert pressure, specifically against escape glycans, on the N- and C-terminal V3 flanks of envelope during long-term HIV-1 infection, wild-type (solid lines) and site-directed mutant (dashed lines) heterologous Envs were pseudotyped and assayed for neutralization with 3-year R880F autologous plasma (**A**). Percent viral infectivity, as adjusted against wells containing no test plasma, is depicted on the vertical axis; reciprocal plasma dilutions are plotted along the horizontal axis in a logarithmic fashion. Each curve represents a single Env-plasma combination, and error bars demonstrate the standard error of the mean of two independent experiments using duplicate wells. In (**B**), the V3/alpha2 helix portion of the labeled Envs has been conceptualized with glycan forks, each of which represents a PNGS in the corresponding amino acid sequences. Glycans of particular interest (N295, N333, and N335) are designated using R880F-specific numbering.

The VH, in particular the CDR H3, has generally been considered a major determinant of epitope recognition and nAb breadth. In our study, VL differences appreciably expanded the neutralization capacity of mAb 19.3H-L3 against autologous Envs. To probe whether this increase in breadth carried over to neutralization of heterologous Envs, mAbs 19.3H-L1 and 19.3H-L3 were tested against a panel of fourteen heterologous Env pseudotypes that included one A/C recombinant, four subtype A, three subtype B, and six subtype C Envs. The mAbs were unable to neutralize any of the heterologous Envs (Figure 9A–B). Thus, while mAb 19.3H-L3 possessed increased breadth against autologous Envs as compared to 19.3H-L1, this did not extend to genetically diverse Envs. Regardless of this restricted mAb cross-clade neutralization, R880F plasma collected at 16-months or 3-years post-infection did have similarly moderate breadth against heterologous Envs, which increased in potency over time (Figure 9C–D). An amino acid alignment of Envs from the heterologous breadth panel demonstrated that Envs neutralized with the greatest potency at 3-years post-seroconversion, A-Q461 and C-Z205F (IC_50_ values of approximately 1∶1000), contained the N335 (HXB2 residue 334) shifted glycan associated with viral escape from mAbs 19.3H-L1 and 19.3H-L3 (Figure 9E). Furthermore, Env A-Q461 also incorporated the N295 (HXB2 residue 293) substitution indicative of mAb escape. To investigate if the N295 glycan addition and/or the shifted N335 glycan in R880F Envs could have been partially responsible for the heterologous neutralization capacity that developed in this subject, several glycan knock-out mutants were created and tested with 3-year R880F plasma (Figure 10A). Within A-Q461, the N295 PNGS was eliminated either alone or in conjunction with the N335 PNGS; the N335 PNGS was also individually knocked out (Figure 10B). The positions of interest were reverted back to the amino acid present in the transmitted/founder Env 0-A6/B24. For C-Z205F, the N335 PNGS was similarly abolished (Figure 10B). Additionally, two heterologous Envs that were only modestly neutralized but that contained the highlighted glycans, C-Z109F and C-Z214M, were mutated as well. All six of the glycan knock-out mutants exactly mirrored their parental equivalents, suggesting that the particular glycans at positions 295 and 335 did not directly contribute to the breadth observed at 3-years post-infection. These data do suggest, however, that early viral escape events likely influenced how breadth developed in this subject, by expanding what was originally a narrow, regional response at the base of the V3 loop to recognize and neutralize distinct portions of Env across genetically diverse variants.

## Discussion

### Initial R880F nAbs target a novel conformational epitope at the base of the V3 domain

Several recent studies detail the nAb responses in early subtype B and C HIV-1 infection [24], [25], [27], [29], [31], [36]. Here we present the first such study of a subtype A infected individual, R880F, where the initial autologous nAb target was defined, along with the consequent routes of viral escape, and two mAbs from early infection were recovered. The kinetics of autologous nAb induction in R880F generally mimicked those described previously for early HIV-1 infection with subtypes A, B, and C [25]–[27], [30], [36], [37]. Reduced neutralizing activity against contemporaneous Envs at each time point indicated a well-established repeating pattern of *de novo* neutralization and viral escape in subject R880F. The early escape Env 2-A3 that differed by only one amino acid residue from the founder Envs, 0-A6 and 0-B24, when combined with a comprehensive panel of mutants, supports the hypothesis that the initial site of nAb recognition was a conformational target at the base of the V3 domain. Specifically, individual mutations at I295, E338, or D341 in R880F conferred escape from 2-month plasma antibodies. The region that encompasses these mutations is close to the gp120 surface area targeted by the broadly neutralizing mAb PGT128 (recovered from a CRF02_AG elite neutralizer) [6], [8], by early plasma nAbs and two mAbs recovered from a subtype B infected seroconverter [25] and by multiple autologous mAbs recovered from two subtype B infected individuals after cessation of antiretroviral treatment [38]. Thus, early nAbs across subtypes commonly target an immunogenic gp120 structure topographically situated near V3, which is well exposed on the Env trimer.

V3-adjacent regions of Env do, nevertheless, elicit strain-specific responses that are easily escaped by multiple pathways. In the study by Bar *et al*., nAbs in one of three subjects (CH40) targeted a putative conformational epitope composed of the same regions bordering V3 that we describe here for R880F. CH40 immune evasion in the V3 flanks was, however, preceded by escape mutations in V1; this suggests that this latter region, also immunogenic in early infection, may have been targeted first [25]. Moore *et al.* recently characterized 2 of 79 subtype C infected subjects who were selected because they developed heterologous plasma neutralization breadth mediated by glycan recognition at HXB2 residue N332, another V3-proximal position. In each of these individuals, the glycan motif at HXB2 residue N334 was present in the founder Env; N332 evolved later as an escape mutation and was subsequently targeted by nAbs [24]. Interestingly, in R880F, the opposite occurred: N332 (R880F residue N333) was present in the founder Env and shifted to N334 (R880F residue N335) as an escape mutation in some Envs. Furthermore, the development of heterologous breadth in R880F was not facilitated by specific recognition of N334 and, therefore, involved additional determinants and complexity. When juxtaposed, these and our studies underscore how identical mutations, when ordered differently during infection, can sometimes drive divergent phenotypic outcomes. Thus, exposure of B cells to a specific sequence of changes in Env can program the course of nAb breadth.

### Select immunoglobulin germline usage emerges in the analysis of HIV-1 nAbs

In our previous study of autologous nAb responses during early subtype C HIV-1 infection in subject Z205F, we reported that multiple mAbs targeted the V1V2 domain [31]. These three Z205F mAbs used somatically related IGHV3-15*01 and IGLV2-14*01 germline gene segments and recognized a series of overlapping conformational epitopes centered on residues N134 in V1 and R189 in V2. Each mAb demonstrated a distinct neutralization profile against early autologous Envs, with variable sensitivity to specific glycans. R880F mAbs similarly utilized a restricted set of IGHV3-30*02 and IGLV2-14*01 germline gene segments, but, in this case, only a single isolated VH exhibited neutralization capacity when paired with the two clonally related VLs named 19.3H-L1 and 19.3H-L3 (Figure S2). In a recent study, a single VH was recovered through phage display and conferred neutralization when paired with four somatically related variants of the same kappa VL [39]. Such VL shuffling produced mAbs with varying neutralizing activities, the most potent of which was dependent on one residue in FWR2 and one residue in CDR3. Moreover, precedent sets of clonally related mAbs that show distinct neutralization potency and/or breadth have been catalogued in HIV-1 infection [6], [7], [15], [38]–[40]. Within the context of our study, it is conceivable that only one R880F VL is authentic, while the other was generated by mutation during short-term *in vitro* stimulation of B cells. This caveat notwithstanding, variation between the neutralizing activities of mAbs 19.3H-L1 and 19.3H-L3 highlights a feasible mechanism for gradual acquisition of autologous breadth against highly related escape variants that was directly attributable to VL changes. Furthermore, in future studies it would be advantageous to recover a greater number of distinct antibodies, as our ability to understand breadth fully here was limited with only two highly related mAbs.

Notably, the mAbs from Z205F and R880F were predicted to utilize the same VL germline, IGLV2-14*01. This germline gene segment is also employed by the broadly neutralizing mAbs PG9 and PG16 that target a quaternary epitope involving V1V2 and V3 and is again paired with a VH3 family gene segment, IGHV3-33*05. These data suggest that VH3 and VL2 pairing is not uncommon for HIV-1 nAbs. Several instances of VH bias for anti-HIV mAbs have been demonstrated based on the epitope: anti-V3 mAbs preferentially use VH5-51 [41], [42]; anti-CD4i mAbs preferentially use VH1-69 [22]; anti-MPER mAbs in more than one instance also utilize VH1-69 [10]; and anti-CD4bs mAbs preferentially use VH1-46 and VH1-2 [13], [15]. These pairings may simply reflect common rearrangement of these germline gene segments in the human immunoglobulin repertoire or the structural features that they bind.

### Structural analysis of mAbs 19.3H-L1 and 19.3H-L3 elucidates antigen-antibody interactions in early HIV-1 infection

Defining the structural characteristics of broadly neutralizing mAbs isolated from elite neutralizers in chronic infection has been a major focus in the HIV-1 nAb field. Unlike the Bar *et al.* study [25], our data here supply structural information regarding HIV-specific mAbs at the opposite end of the neutralization spectrum. Indeed, we are among the first to report high-resolution crystal Fab structures from early HIV-1 infection, and to show that these mAbs likely mediate planar interactions with antigen that can be subtly altered by VL changes. Structural analyses of the 19.3H-L1 and 19.3H-L3 antigen-binding sites are consistent with the neutralization data that place their epitopes at the base of the V3 domain. As this region of gp120 lies flat, any one of the three single amino acid changes that conferred escape at 2-months could potentially disrupt the planar interactions between the 19.3H-L1 and 19.3H-L3 antigen-binding sites and their epitopes, as discussed below.

Introducing a positively charged residue with a long side chain (I295R) or a glycan (I295N) at position 295 is not compatible with the flat hydrophobic surface of the 19.3H-L1/19.3H-L3 antigen-binding site. In fact, neither mAb could bind to monomeric R880F gp120 containing the I295R mutation. In this model, the I295T substitution would be less effective at conferring neutralization escape. The long, negatively charged E338 side chain is predicted to interact with one of the positively charged surface patches (Figure 7C, blue) at the edge of the 19.3H-L1/19.3H-L3 antigen-binding site, potentially forming a salt bridge with the side chain of a positively charged residue there. The E338K mutation probably destroys this interaction and creates an electrostatic repulsion, which is also consistent with the lack of mAb binding to monomeric R880F gp120 containing the E338K mutation. Interestingly, E338D at this position does not allow 19.3H-L1 and 19.3H-L3 to neutralize the viruses, suggesting that the length of the Asp side chain is not sufficiently long to restore the possible salt bridge. These results suggest that both length and negative charge of the side chain at E338 are important for antibody binding.

The highly conserved N333 (HXB2 residue 332) PNGS at the base of V3 is located at the edge of the proposed epitope and potentially interacts with 19.3H-L1 and 19.3H-L3, as removal of this glycan (S335Q) weakens the neutralization capacities of these two antibodies, most dramatically in the case of 19.3H-L1. Moreover, the glycan shift from position 333 to 335 (S335N), toward the center of the epitope, also prevents the flat-surface antigen-antibody interaction. In combination, the structural, neutralization, and ELISA binding data indicate that mAbs 19.3H-L1 and 19.3H-L3 likely recognize overlapping epitopes that are centered on I295 and E338; however, 19.3H-L1 is more dependent on D341N and the N333 glycan motif for neutralization than 19.3H-L3. Wholly, these analyses suggest that planar motifs that lie across a flat antigen surface could mediate antibody-antigen recognition in early HIV-1 infection, prior to multiple rounds of viral escape and perhaps more extensive affinity maturation. Additionally, the specific determinants for optimal antigen recognition by each mAb, and the strengths of R880F founder Env gp120 binding, differ slightly as a result of VL variation.

### Sequential exposure to certain patterns of Env escape could program humoral immunity for the development of nAb breadth

In most cases, neutralization breadth in chronic infection has been attributed to the VH, with particular emphasis on the CDR H3 [8], [22], [43]–[45]. Few studies have, however, investigated the roots of neutralization breadth, as was done here. We found, somewhat unexpectedly, that in R880F, VL sequence variation influenced mAb 19.3H-L3's ability to neutralize two autologous escape variants that were not neutralized by mAb 19.3H-L1 during early infection. Significant augmentation of autologous neutralization via minor VL variation (instead of extensive CDR H3 lengthening) supports a potential mechanism for how escape variants that differ by only a few amino acids and/or glycans are neutralized. Based on this, we contend that the maintenance of VH-determined epitope specificity while light chain antigen contacts are varied could represent an important breadth-augmenting mechanism for B cells responding to highly related Env escape variants. More dramatic nAb structural adaptations such as the elongation of CDR H3 may require time for development, as longitudinal viral variants establish more complex ploys to escape.

Collectively, several factors appeared to shape the antibody maturation pathways in R880F: (i) the initial site of nAb recognition, (ii) VH and VL rearrangement, pairing, and somatic hypermutation, and (iii) repeated exposure to highly related Env escape variants. Our data are consequently consistent with the idea that neutralization breadth arises through the sequential exposure of somatically related B cells to a cascade of viral escape variants presenting altered versions of the same epitope. Additionally, and in contrast to the Moore *et al.* report [24], our findings demonstrate that glycans, which arose in response to the initial waves of neutralization, do not always become subsequent targets for later nAbs or promote the potential to develop heterologous breadth. Moving forward, better understanding of how initial immunoglobulin targeting affects downstream neutralization potential could positively impact HIV-1 vaccine design. Our studies suggest that the mere presence of a PNGS does not ensure its recognition by an antibody. Sequential exposure to glycans and other Env variations may be required to drive the type of specialized antibody response associated with elite neutralization. In fact, support for this type of immunization approach has been demonstrated [46]. It is, however, currently unknown exactly how to accelerate somatic hypermutation, lengthening of the CDR H3, or the acquisition of other adaptations that lead to increased breadth. We propose that a viable vaccination strategy may involve immunizing with a carefully selected series of Env immunogens that mimic defined amino acid and/or PNGS changes that occurred during the natural viral escape process and led to increased neutralization breadth, such as those described here.

## Materials and Methods

### Ethics statement

Both the Emory University Institutional Review Board and the Rwanda Ethics Committee approved informed consent and human subjects protocols, and subject R880F provided written informed consent for study participation.

### Study subject R880F (IAVI 00C175038)

Longitudinal plasma and PBMC samples were obtained from ART-naïve subject R880F during enrollment in International AIDS Vaccine Initiative (IAVI) Protocol C at Projet San Francisco (PSF) in Kigali, Rwanda, as part of a multi-site study of early HIV-1 infection in adult Africans. The PSF cohort, which provides voluntary HIV-1 testing, counseling, and condom provision to cohabiting heterosexual couples, is discussed in more comprehensive detail in [47], [48]. Plasma viral load determination (reported in Table 1) was underwritten by IAVI and performed at Contract Lab Services (CLS) in South Africa using an Abbott m2000 system where typical detection ranged between 160 and 4×10^7^ copies/ml.

### Amplification, cloning, and function screening of HIV-1 env genes from R880F

Conditions for plasma viral RNA extraction and purification, cDNA synthesis, and nested single-genome PCR amplification have been described previously [49]. Subsequent full-length Env gp160 coding regions (plus Rev, Vpu, and partial Nef) were TA cloned into the CMV promoter-driven expression plasmid pcDNA3.1/V5-His-TOPO (Invitrogen) and screened for biological function as pseudoviruses following co-transfection with an Env-deficient subtype B proviral plasmid (pSG3Δenv) in 293T cells using FuGENE HD (Roche or Promega). Forty-eight hours later, supernatant was collected, clarified at 3,000 rpm for 20 min, and used to infect Tzm-bl cells. Following another 48-hour incubation, β-gal staining was performed, and wells were scored positive or negative for blue foci.

### Envelopes used for heterologous breadth screen

Fourteen subtype A, B, and C envelopes were used to evaluate the heterologous neutralization breadth of R880F mAbs 19.3H-L1 and 19.3H-L3 along with autologous 16-month and 3-year plasmas. One A/C recombinant and three subtype C early transmitted variants were previously cloned in our laboratory, as described in [49]: A/C-R66M is R66M 7Mar06 3A9env2; C-Z205F is Z205F 27Mar03 (“0-month”) EnvPL6.3 [29], [31]; C-Z1792M is Z1792M 18Dec07 3G7env2; and C-Z185F is Z185F 24Aug02 (“0-month”) EnvPB3.1 [29]. Ten envelopes were obtained through the AIDS Research and Reference Reagent Program, Division of AIDS, NIAID, NIH: from Dr. Julie Overbaugh, A-Q769-b9 is Q769_ENVb9_ (Cat#11545), A-Q769-d22 is Q769_ENVd22_ (Cat#10458), A-Q461 is Q461_ENVd1_ (Cat#11544), and A-Q23 is Q23_ENV17_ (Cat#10455) [50]–[52]; from Drs. David C. Montefiori, Feng Gao, and Ming Li, B-SS1196 is SS1196.1 (Cat#11020), and B-TRO is TRO, clone 11 (Cat#11023) [53]; from Drs. Beatrice H. Hahn, Yingying Li, and Jesus F. Salazar-Gonzalez, B-gp160opt is pConBgp160-opt (Cat#11402), C-gp160opt is pConCgp160-opt (Cat#11407), and C-Z214M is ZM214M.PL15 (Cat#11310) [54]–[56]; and from Drs. Cynthia A. Derdeyn and Eric Hunter, C-Z109F is ZM109F.PB4 (Cat#11314) [57].

### PCR-based site-directed mutagenesis

Mutations were created through PCR using two overlapping primers that contained the mutated sequence per Env, in a strategy similar to that described previously [29], [31], [58], [59]. Briefly, the plasmids containing 0-A6, 0-B24, 2-A3, 2-B12, 5-B52, A-Q461, C-Z205F, C-Z109F, or C-Z214M *env* genes were amplified with the following sets of forward (F) and reverse (R) primer sequences, where the mutated nucleotides are underlined:

For mutants 0-A6 I295N and 2-B12 I295N: F 5′-cagcctgtgaatattacgtgtattagaactggc-3′ and R 5′-gccagttctaatacacgtaatattcacaggctg-3′

For mutant 0-B24 I295R: F 5′-gcccagcctgtgagaattacgtg-3′ and R 5′-cacgtaattctcacaggctgggc-3′

For mutant 0-B24 I295T: F 5′-cttgcccagcctgtgacaattacgtgtattag-3′ and R 5′-ctaatacacgtaattgtcacaggctgggcaag-3′

For mutants 0-A6 S335N and 2-B12 S335N: F 5′-gcatattgtaatgtcaatagaacagaatgg-3′ and R 5′-ccattctgttctattgacattacaatatgc-3′

For mutant 5-B52 S335N: F 5′-gcatattgtaatgtcaatagaacaggatgg-3′ and R 5′-ccatcctgttctattgacattacaatatgc-3′

For mutant 2-B12 S335Q: F 5′-gcatattgtaatgtccaaagaacagaatgg-3′ and R 5′-ccattctgttctttggacattacaatatgc-3′

For mutant 2-A3 K338D: F 5′-gtcagtagaacagactggaatgacactttac-3′ and R 5′-gtaaagtgtcattccagtctgttctactgac-3′

For mutant 2-A3 K338G: F 5′-gtcagtagaacaggatggaatgacactttac-3′ and R 5′-gtaaagtgtcattccatcctgttctactgac-3′

For mutant 2-A3 K338G D341N: F 5′-gtcagtagaacaggatggaatgacactttac-3′ and R 5′-gtaaagtgtcattccatcctgttctactgac-3′ followed by F 5′-gtagaacaggatggaataacactttacaacaggtag-3′ and R 5′-ctacctgttgtaaagtgttattccatcctgttctac-3′

For mutant 2-A3 K338I: F 5′-gtcagtagaacaatatggaatgacactttac-3′ and R 5′-gtaaagtgtcattccatattgttctactgac-3′

For mutant 2-A3 K338Q: F 5′-gtcagtagaacacaatggaatgacactttac-3′ and R 5′-gtaaagtgtcattccattgtgttctactgac-3′

For mutant 2-A3 K338R: F 5′-gtcagtagaacaagatggaatgacactttac-3′ and R 5′-gtaaagtgtcattccatcttgttctactgac-3′

For mutant 0-B24 D341N: F 5′-cagaatggaataacactttacaacagg-3′ and R 5′-cctgttgtaaagtgttattccattctg-3′

For mutant 0-B24 E456K: F 5′-gagatggtggtaaggatattaacag-3′ and R 5′-ctgttaatatccttaccaccatctc-3′

For mutant A-Q461 N295I: F 5′-ccaagcctgtgataattacttgtatcagacctggc-3′ and R 5′-gccaggtctgatacaagtaattatcacaggcttgg-3′

For mutant A-Q461 N335S: F 5′-gcacattgtgttgtcagtagaacagagtggaataac-3′ and R 5′-gttattccactctgttctactgacaacacaatgtgc-3′

For mutant A-Q461 N295I N335S: F 5′-ccaagcctgtgataattacttgtatcagacctggc-3′ and R 5′-gccaggtctgatacaagtaattatcacaggcttgg-3′ followed by F 5′-gcacattgtgttgtcagtagaacagagtggaataac-3′ and R 5′-gttattccactctgttctactgacaacacaatgtgc-3′

For mutant C-Z205F N335S: F 5′-caagcatattgtagcattagtaaaagtaaatggaatgac-3′ and R 5′-gtcattccatttacttttactaatgctacaatatgcttg-3′

For mutant C-Z109F N335S: F 5′-gaaaggcatattgtaaaattagtggaagtgagtggaatg-3′ and R 5′-cattccactcacttccactaattttacaatatgcctttc-3′

For mutant C-Z214M N295I: F 5′-caacttacagaagctgtaataattacgtgtatgaggccc-3′ and R 5′-gggcctcatacacgtaattattacagcttctgtaagttg-3′

The PCR cycling parameters were 1 cycle of 95°C for 2 min; 30 cycles of 95°C for 20 sec, 50°C to 60°C for 20 sec (the optimal annealing temperature was determined for each primer set), and 72°C for 2 min; and 1 cycle of 72°C for 3 min. The samples were then stored at 4°C. The 25-µl PCR mixtures contained 62.5–250 ng of each primer, 5–30 ng of the plasmid template, 0.4 mM dNTPs, and 1× reaction buffer. PfuUltra II Fusion HS DNA Polymerase (Stratagene) was used to generate the PCR amplicons, which were digested with 20 U *DpnI* (NEB) for 1 hr to remove contaminating template DNA and then transformed into maximum-efficiency XL10-Gold ultracompetent cells (Stratagene) so that the DNA volume did not exceed 5% of the cell volume. Transformed cells were plated onto LB-ampicillin agar plates, which generally resulted in 50 to 100 colonies. Isolated colonies were inoculated into LB-ampicillin broth for overnight shaking (225 rpm at 37°C), and plasmids were purified using a QIAprep spin miniprep kit (Qiagen). All *env* mutations were confirmed by nucleotide sequencing.

### DNA sequencing and analysis

Sanger DNA sequencing of wild-type and mutant envelope genes and immunoglobulin genes was executed with an ABI 3730xl DNA Analyzer and BigDye Terminator v3.1 chemistry at one of two facilities: the University of Alabama at Birmingham Center for AIDS Research (P30-A127767) DNA Sequencing Shared Facility or GenScript. Nucleotide sequences were edited and assembled using Sequencher v5.0 and deposited into GenBank under accession numbers JX096639-JX096660 for wild-type *env* clones and JX124277-JX124282 for immunoglobulin genes. Amino acid sequences were translated and aligned using Geneious v5.0.3.

### Neutralization assay

Five-fold serial dilutions of heat-inactivated R880F plasma samples, antibody-containing 293T supernatants, or purified R880F monoclonal antibodies were assayed for neutralization potential against viral pseudotypes in the Tzm-bl indicator cell line, with luciferase as the ultimate readout, as described previously [29], [37], [57], [58], [60]. In short, Tzm-bl cells were plated and cultured overnight in flat-bottomed 96-well plates. Pseudovirus (2,000 IU) in DMEM with ∼3.5% FBS (HyClone), 40 µg/ml DEAE-dextran was incubated with serial dilutions of plasma, supernatant, or antibody, and subsequently, 100 µl was added to the plated Tzm-bls for a 48 hr infection before being lysed and evaluated for luciferase activity. Data was retrieved from a BioTek Synergy HT multi-mode microplate reader with Gen 5, v1.11 software, the average background luminescence from a series of uninfected wells was subtracted from each experimental well, infectivity curves were generated using GraphPad Prism v4.0c where values from experimental wells were compared against a well containing virus only with no test reagent, and IC_50_ values were determined using linear regression in Microsoft Excel for Mac 2011, v14.0.2.

### Homology modeling of Env V3-proximal residues 295, 338, and 341

The subject R880F 0-B24 Env gp120 sequence was modeled using the MODELLER program [61]. The template for the homology model was a subtype A gp120 obtained by longtime all-atom molecular dynamics simulation using the CHARMM27 potential in the NAMD program [62]. This simulated gp120 was modeled using all known CD4-bound gp120 structures (Protein Data Bank [PDB] accession numbers 1G9M [63],1RZK [22], 2B4C [64], 2NY7 [65], 3JWD and 3JWO [66], and 3LMJ [43]) as templates. In all of these structures, the core of gp120 was highly similar; however, it should be noted that none of these structures is subtype A. Multiple templates were used because it has been shown that this creates high quality homology models. In addition, each template has slightly different regions of gp120 resolved. Before modeling, the templates were arranged in the trimeric state, which has been resolved using cryoelectron microscopy (PDB accession number 3DNO [67]), to ensure that the hypervariable loops did not sterically clash with the neighboring monomers. During modeling, disulfide constraints were added for the conserved cysteines present in all gp120 sequences. All sequence alignments used for modeling templates were based on sequences in the HIV-1 database (www.hiv.lanl.gov).

### Modeling of Env V3-proximal glycans at residues 295 and 335

The subject R880F 0-B24 Env gp120 sequence mutated with N295 and N335 was modeled using the protocol described in [31]. Xleap in AmberTools kit 1.4 was used to add two glycans at residues N295 and N335. A five-mannose glycan was used in this simulation because it was found to be the most abundant glycan form in the immunodeficiency virions [68]. Amber99SB force field [69] was used for the gp120 protein, and GLYCAM06 force field [70] was used for the five-mannose glycan. All the systems were minimized using a 3-step protocol in which the protein was gradually allowed to move. These steps were: heavy atoms fixed (5,000 steps), protein backbone atoms fixed (5,000 steps), and all atoms free to move (20,000 steps). The system was gradually heated in a four-step process. The initial temperature was set to 100 K, and only hydrogen atoms were allowed to move for 25,000 fs. In the second step, the temperature was set at 300 K, and heavy atoms in the protein were harmonically constrained for the next 25,000 fs. Then the temperature was raised to 500 K, and backbone atoms were harmonically constrained for 25,000 fs. Force constants for all harmonic constraints were set to 1 kcal mol^−1^Å^−2^. Finally, the temperature was raised to 700 K, and the backbone atoms in the core of gp120 were constrained for the next 4.925 ns. The coordinates were saved once every ps, in these 5 ns. The MD simulation was performed using NAMD 2.8 [62]. The conformation at the end of the 5 ns MD simulation was used in this study.

### Cloning of immunoglobulin VH and VL genes from R880F B cells

A viably frozen PBMC sample from subject R880F was collected at 16-months post-infection and was used to recover autologous mAbs 19.3H-L1 and 19.3H-L3. The first phase of recovery was performed in the Robinson laboratory. Non-B cells were depleted using immunomagnetic beads (Miltenyi). Approximately 100,000 B cells were recovered, and for memory B cell stimulation, the cultures were incubated for 3 days in RPMI medium containing 10% FCS, Epstein-Barr virus, 2 µg/ml R848 (InVivogen), and 100 U/ml IL-2 (Dr. Maurice Gately, Hoffmann - La Roche Inc. [71]). The cells were then plated into 3,840 wells in the same medium at low cell densities (30 to 50 cells/well) in forty 96-well tissue culture plates containing irradiated macrophage or human placental fibroblast feeder cells. Starting at 12 days of culture, B cell culture supernatants were screened every 3 to 4 days for antibodies that neutralized the autologous founder pseudovirus containing Env 0-B24, or that showed ELISA reactivity with 0-B24 Env glycoproteins in previously described assays [31], [38]. Supernatant from 63 wells screened positive for inhibitory activity in the Tzm-bl assay, and 35 of these were also positive for gp120 binding activity in ELISA. The positive cultures were placed in RNAlater between days 17 to 21 after stimulation. RNA was purified from 12 lysates of these B cell cultures that had been found to be antibody positive. RNA from each well was reversed transcribed into cDNA encoding VH and lambda/kappa VL genes, which were then amplified in a nested PCR as described by Liao *et al*. [72]. VH and VL gene products were assembled by overlapping PCR into pairs of linear expression vectors encoding full-length human Ig heavy and light chain genes [72]. These vectors were co-transfected into wells containing 80–90% confluent 293T cells. Two days later, supernatants of transfected cultures were tested for antibody activity in the same assays used to screen B cell cultures. One well of 293T cells transfected with VH and VL genes originating from a single B cell culture designated 19.3H (plate 19, well 3H) was found to be antibody positive. The antibody (or antibodies) produced was thus named 19.3H. 293T cells expressing antibody 19.3H were serially passaged at limiting cell densities under blasticidin selection (for maintenance of the Ig vector) to obtain multiple clones. Selected 19.3H-derived clones were expanded into stable antibody producing cell lines to facilitate purification of the antibody by Protein A affinity chromatography.

To obtain VH and VL sequences that corresponded to the 19.3H antibody activity, VH and VL genes were isolated from the selected 19.3H-derived 293T cell clones using two different methods in the second phase of recovery. The first method was used in the Robinson laboratory. VH and VL genes were re-amplified from the selected 293T cell clones and inserted into expression plasmids obtained from InVivoGen: pFUSE-CHIg-hG1, containing the constant region of the human IgG1 heavy chain, and pFUSE2-CLIg-hl2 containing the constant region of human Ig lambda 2 light chain, respectively. First, the pFuse vectors were linearized by digestion with *EcoRI* and then subjected to PCR with primers (IgVH FWD 5′-CGAACCGGTGACGGTGTCGTGGAAC-3′ and REV 5′-ACCGGTGATCTCAGGTAGGCGCC-3′, IgVLambda FWD 5′-CCAACAAGGCCACACTGGTGTGTCTC-3′ and REV 5′-ACCGGTGATCTCAGGTAGGCGCC-3′, IgVKappa FWD 5′-GAACTGCCTCTGTTGTGTGCCTGCTG-3′ and REV 5′-ACCGGTGATCTCAGGTAGGCGCC-3′) to generate annealing sites of 15 nucleotides that were homologous with ends of the inserts [73]. Second, the SuperScript III One-Step RT-PCR System (Invitrogen) was used to amplify the Ig variable regions from 293T-cell-derived mRNA using primers designed to synthesize inserts for use with the ligation-independent In-Fusion cloning system (Clontech). The forward primer IgVH,IgVLambda,IgVKappa FWD 5′-CCTGAGATCACCGGTGCTAGCACCATGGAGACAGACACACTCC-3′ was used for both heavy and light chain inserts and contained a non-annealing tag with 15 nucleotides of homology to the upstream insertion site on the plasmid. Reverse primers for each heavy and light chain (IgVH REV 5′-CACCGTCACCGGTTCGGGGAAGTAG-3′, IgVLambda REV 5′GTGTGGCCTTGTTGGCTTGAAGCTCCTC-3′, IgVKappa REV 5′-CACAACAGAGGCAGTTCCAGATTTCAACTGCTC -3′) contained 15–20 nucleotides that overlapped with the 5′ end of the constant regions in linearized pFuse vectors. The In-Fusion reaction was performed according to manufacturer's instructions. Plasmids containing inserts were grown in JM109 competent cells, and at least five colonies were picked for subsequent nucleotide sequencing.

A second approach was performed in the Derdeyn laboratory to recover the VH and VL genes from the 19.3H-derived 293T cell clones, and from In-Fusion plasmids generated in the Robinson lab, such that all VH and VL genes would be expressed from the same plasmid vector for the neutralization studies. PCR of VH and lambda/kappa VL genes was performed essentially as described by [74], [75]. Briefly, nested PCR was performed using PfuUltra II Fusion HS DNA Polymerase (Stratagene) using the primers described. The first round amplified the leader to constant regions of the VH and VL genes, using cDNA from a 19.3H-derived 293T clonal cell line or In-Fusion plasmid DNA as a template. The second round PCR was performed to amplify the variable regions. PCR products were gel purified, digested with appropriate enzymes (*AgeI* and *SalI* for VH, *AgeI* and *XhoI* for VL, all enzymes from NEB), and cloned into the plasmid expression vectors kindly provided by Dr. Patrick Wilson (heavy - accession number FJ475055, lambda - accession number FJ517647). Plasmids were grown in One Shot TOP10 chemically competent *E. coli* cells (Invitrogen) and purified with a QIAprep spin miniprep kit (Qiagen). At least three separate colonies were picked and sequenced. In the end, one VH and two somatically related lambda VL genes were recovered from five 19.3H-derived 293T clonal cell lines. The VH combined with either of the VLs (but not randomly with VLs from other R880F B cell cultures) produced robust neutralizing activity against the R880F founder Envs 0-A6 and 0-B24. Further characterization of the mAbs against the larger panel of R880F Envs revealed that the VLs had distinct neutralizing capacities when combined with the 19.3H VH, but no neutralizing activity when combined randomly with R880F VHs from other B cell wells. The mAbs containing the different VLs were then designated 19.3H-L1 and 19.3H-L3.

### Production of monoclonal antibodies

293T cells were cultured in T-75 flasks in DMEM with 10% FBS until 80% confluency was reached. Equal amounts (6 µg) of VH- and VL-containing plasmids were mixed with FuGENE HD (Roche) at a 1∶3 ratio and used for transfection. After 24 hr, media was removed, cells were washed twice with PBS, and the media was replaced with basal media (DMEM, 1% PSG, 1% Nutridoma SP). Cells were incubated for four days at 37°C, after which the supernatant was harvested. Cell debris was removed by centrifugation at 1,500 rpm for 5 min. Approximately 50 ml culture supernatant was used for antibody purification using a Protein A/G Spin column (Pierce) according to manufacturer's instructions. Purified antibodies were concentrated using Vivaspin concentrators (GE), and protein concentrations were determined using a Nanodrop spectrophotometer (BioTek).

### Biotinylation of monoclonal antibodies

Four monoclonal antibodies were biotinylated with the EZ-Link Sulfo-NHS-LC-Biotinylation Kit (Thermo Scientific) for use in ELISA protocols: 19.3H-L1 and 19.3H-L3 isolated here from R880F, 6.4C isolated from Z205F [31], and PGT128 obtained through the IAVI Neutralizing Antibody Consortium (NAC) Protocol G mAb Reagent Program [6], [76]. For each mAb, 50 µg were diluted in 500 µl 1× PBS (0.1 M sodium phosphate, 0.15 M NaCl, pH 7.2) for a final protein concentration of 100 µg/ml. A 50-fold molar excess of biotin was incubated with each mAb for 1 hour at room temperature. Excess biotin was removed via Zeba Desalt Spin Column, per the manufacturer's instructions.

### gp120 binding and competition ELISAs

Reacti-Bind polystyrene 96-well plates (Thermo Scientific) were coated overnight at 4°C with 100 µl/well of 2 µg/ml R880F 0-A6/B24, R880F 0-A6/B24 I295R, or R880F 0-A6/B24 E338K purified gp120 protein (Life Technologies, GeneArt) in PBS. Note that blank control wells were coated with gp120 protein but were never subjected to mAb incubation to determine background absorbance, which averaged at 0.055, and assays were performed in duplicate. Plates were subsequently washed six times with 1× PBS-T (Thermo Scientific; 10 mM sodium phosphate, 0.15 M NaCl, 0.05% Tween-20) and blocked with 200 µl/well of 1× B3T buffer (150 mM NaCl, 50 mM Tris-HCl, 1 mM EDTA, 3.3% FBS, 2% BSA, 0.07% Tween-20) for 1 hour at 37°C in a CO_2_-free incubator. During this incubation step, a two-fold dilution series that spanned 11 wells was prepared in 1× B3T for each biotinylated mAb (19.3H-L1, 19.3H-L3, PGT128, or the negative control, 6.4C) to be tested for binding, beginning at a concentration of 10 µg/ml. Plates were washed six times with 1× PBS-T a second time, and 100 µl/well of serially-diluted mAb was incubated for 1 hour at 37°C. Plates were washed six times with 1× PBS-T a third time, and 100 µl/well of a 1∶10,000 dilution of high sensitivity streptavidin horseradish peroxidase (HRP) conjugate (Thermo Scientific) in 1× B3T was incubated for 1 hour at 37°C. After a final six-time wash with 1× PBS-T, 100 µl of room temperature SureBlue 3,3′,5,5′ tetramethylbenzidine (TMB) microwell peroxidase substrate solution (KPL) was added to each well and incubated for 5 minutes at room temperature. To cease colorimetric development, 100 µl/well of 2 M H_2_SO_4_ was added, and absorbance values at 450 nm were read with a BioTek Synergy HT multi-mode microplate reader. Data was retrieved with KC4 v3.4 software, and binding curves were generated using GraphPad Prism v5.0d.

The gp120 binding ELISA protocol was minimally modified to measure the competitive binding of multiple mAbs, via the following alterations: Only R880F 0-A6/B24 gp120 protein was used, and PGT128 was excluded from the competitions. The first of two 100 µl/well mAb incubation steps was performed via a three-fold dilution series that spanned 7 wells; here, each mAb to be tested for competition (19.3H-L1, 19.3H-L3, or the negative control, 6.4C) was prepared in 1× B3T, beginning at a concentration of 10 µg/ml. The second of two 100 µl/well mAb incubation steps involved addition of a constant 1 µg/ml biotinylated competitor (either 19.3H-L1 or 19.3H-L3) across all wells. Wells were washed six times with 1× PBS-T between these two 1 hour, 37°C incubations. To determine 100% binding for 1 µg/ml biotinylated 19.3H-L1, 19.3H-L3, and 6.4C, duplicate wells were incubated with 1× B3T only during the first mAb incubation step and the appropriate biotinylated competitor during the second. The average absorbance for biotinylated 6.4C alone was 0.056. Background absorbance averaged at 0.048.

### Determination of Fab crystal structures

Fab fragments of monoclonal antibodies 19.3H-L1 and 19.3H-L3 were crystallized using previously described methods [41], [77]–[79]. In short, Fab fragments were generated by papain digestion, purified using affinity and size exclusion chromatography, concentrated, and crystallized with the hanging drop method. Fab 19.3H-L1 was crystallized with a well solution containing 0.17 M (NH4)_2_SO_4_, 0.085 M cacodylate pH 6.5, 25.5% (w/v) polyethylene glycol (PEG) 8000, and 15% (v/v) glycerol. Fab 19.3H-L3 was crystallized with a well solution containing 28% PEG 4K, 0.17 M Li_2_SO_4_, 0.085 M Tris pH 8.5, and 15% glycerol. X-ray diffraction data were collected at beamline 23-ID-D GM/CA-CAT at the Advanced Photon Source of Argonne National Laboratory, and the data sets were processed using HKL2000 [80]. Crystal structures were solved by the molecular replacement method using MOLREP in CCP4 [81], [82]. A homologous Fab (PDB code 3NH7) was used as the starting model. The structures were refined using COOT [83] and PHENIX [84], and analyzed using ICM [85]. The Protein Data Bank (http://www.rcsb.org/pdb) accession numbers for the coordinates of the structures of Fabs 19.3H-L1 and 19.3H-L3 are 4F57 and 4F58, respectively.

## Supporting Information

Figure S1 **Full-length R880F Env gp160 alignment.** The complete Env gp160 amino acid sequences of twenty longitudinal R880F humoral escape variants have been aligned in comparison to the founder Envs 0-A6/B24 and examined using Sequencher v5.0 and Geneious v5.0.3 software. Dashes represent conserved positions; dots represent gaps. Significant structural domains are highlighted for simple visual discernment (V1 = red, V2 = blue, V3 = magenta, alpha2 helix = green, V4 = orange, V5 = purple).
(TIF)

Figure S2 **Specificity of R880F VH and VL pairing.** 19.3H-HC, a common R880F heavy chain shared by the two R880F mAbs, was stochastically paired and co-transfected with two unrelated R880F light chains, 15.1B-LC2 (**A**) and 15.10G-LC2 (**B**). Similarly, the wild-type 19.3H-L3 light chain was matched with a random R880F heavy chain, 3.11A-HC1 (**C**). All chimeric mAbs were examined for neutralization capacity against a panel of ten R880F longitudinal Envs (0-month Env = circle, 2-month Envs = triangles, 5-month Envs = inverted triangles, 7-month Env = square, 10-month Env = diamond). Percent viral infectivity, as adjusted against wells containing no supernatant, is depicted on the vertical axis; supernatant dilutions are plotted along the horizontal axis in a logarithmic fashion. Each curve represents a single Env-supernatant combination, and error bars demonstrate the standard error of the mean of two independent experiments using duplicate wells. Supernatant from the co-transfection of 19.3H-HC with wild-type light chain 19.3H-L3 was used as a positive control in these experiments (**D**). Germline heavy and light chain gene segment utilization was determined by SoDA, a somatic diversification analysis program [32], and amino acid sequences were aligned and examined using Sequencher v5.0 and Geneious v5.0.3 software (**E**). Dashes represent conserved positions.
(TIF)

Table S1 **Fab crystal structure data collection and refinement statistics.** Fab fragments of R880F mAbs 19.3H-L1 (PDB code 4F57) and 19.3H-L3 (PDB code 4F58) were crystallized with the hanging drop method after papain digestion, affinity and size exclusion chromatography purification, and concentration. X-ray diffraction data were collected, processed using HKL2000, and are shown in the top half of the table. Statistics in parentheses in the 19.3H-L1 and 19.3H-L3 columns refer to outer shell resolutions. The structures were refined using COOT and PHENIX and analyzed using ICM; these values are shown in the bottom half of the table.
(DOCX)
