# Supplementary figures and images for: Viral Escape from Neutralizing Antibodies in Early Subtype A HIV-1 Infection Drives an Increase in Autologous Neutralization Breadth
Source: PLoS Pathog. 2013 Feb 28;9(2):e1003173. doi: 10.1371/journal.ppat.1003173 (PMC3585129; doi:10.1371/journal.ppat.1003173)

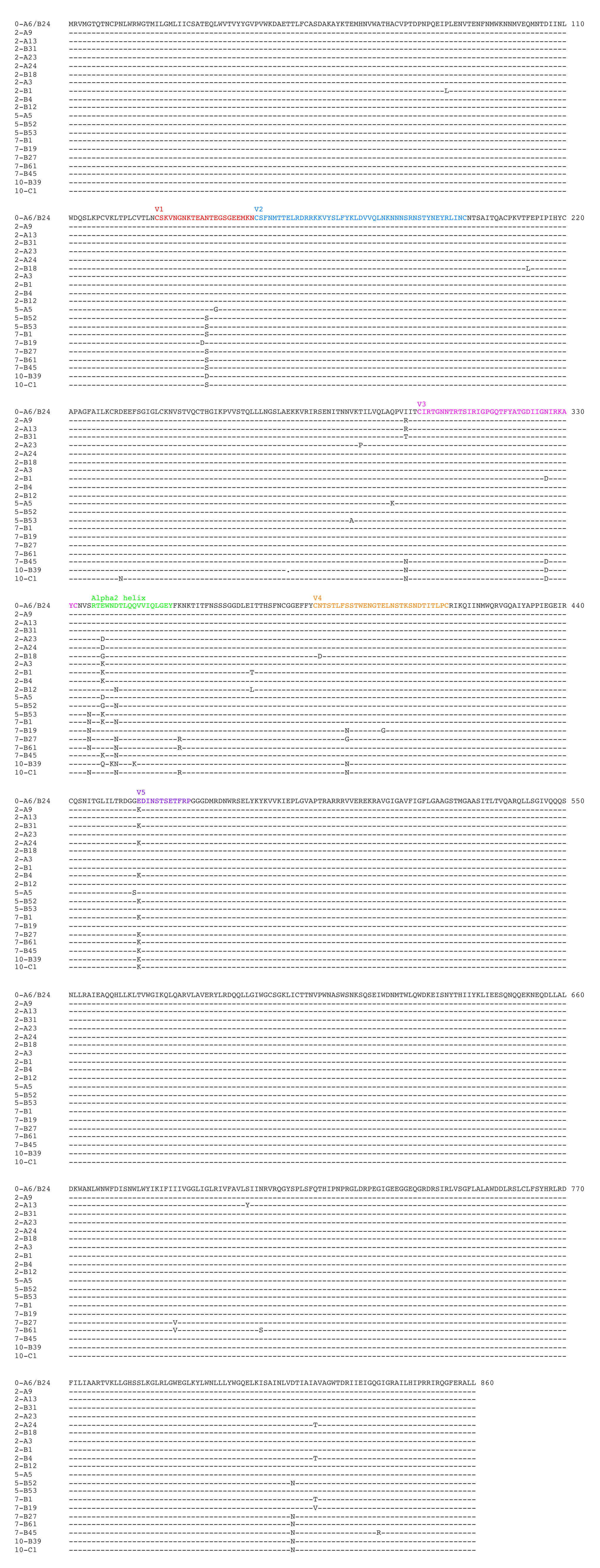

Supplement: Figure S1 — Full-length R880F Env gp160 alignment. The complete Env gp160 amino acid sequences of twenty longitudinal R880F humoral escape variants have been aligned in comparison to the founder Envs 0-A6/B24 and examined using Sequencher v5.0 and Geneious v5.0.3 software. Dashes represent conserved positions; dots represent gaps. Significant structural domains are highlighted for simple visual discernment (V1 = red, V2 = blue, V3 = magenta, alpha2 helix = green, V4 = orange, V5 = purple). (TIF) [file ppat.1003173.s001.tif]

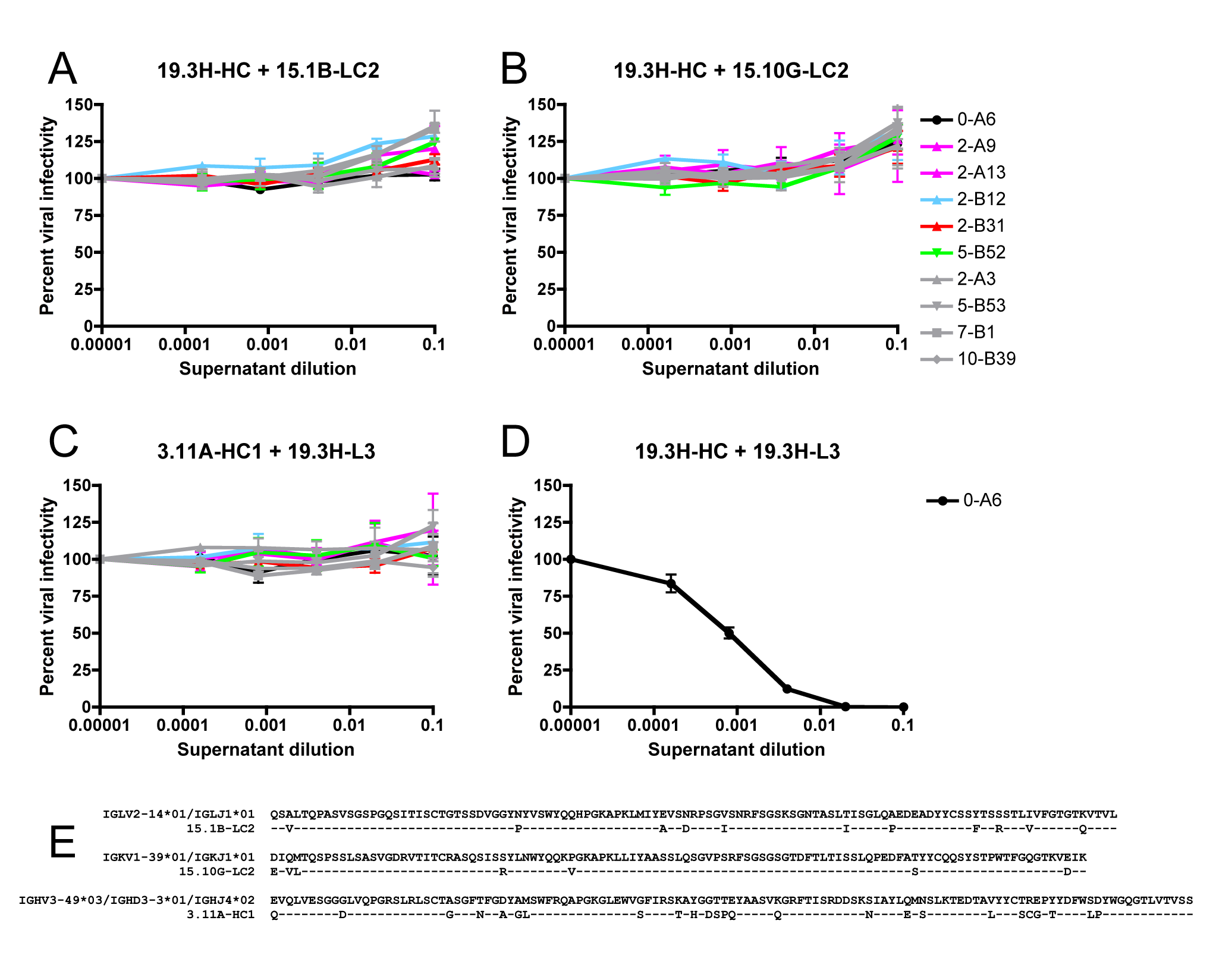

Supplement: Figure S2 — Specificity of R880F VH and VL pairing. 19.3H-HC, a common R880F heavy chain shared by the two R880F mAbs, was stochastically paired and co-transfected with two unrelated R880F light chains, 15.1B-LC2 (A) and 15.10G-LC2 (B). Similarly, the wild-type 19.3H-L3 light chain was matched with a random R880F heavy chain, 3.11A-HC1 (C). All chimeric mAbs were examined for neutralization capacity against a panel of ten R880F longitudinal Envs (0-month Env = circle, 2-month Envs = triangles, 5-month Envs = inverted triangles, 7-month Env = square, 10-month Env = diamond). Percent viral infectivity, as adjusted against wells containing no supernatant, is depicted on the vertical axis; supernatant dilutions are plotted along the horizontal axis in a logarithmic fashion. Each curve represents a single Env-supernatant combination, and error bars demonstrate the standard error of the mean of two independent experiments using duplicate wells. Supernatant from the co-transfection of 19.3H-HC with wild-type light chain 19.3H-L3 was used as a positive control in these experiments (D). Germline heavy and light chain gene segment utilization was determined by SoDA, a somatic diversification analysis program [32], and amino acid sequences were aligned and examined using Sequencher v5.0 and Geneious v5.0.3 software (E). Dashes represent conserved positions. (TIF) [file ppat.1003173.s002.tif]
