# Supplementary material for: Viral Escape from Neutralizing Antibodies in Early Subtype A HIV-1 Infection Drives an Increase in Autologous Neutralization Breadth
Source: PLoS Pathog. 2013 Feb 28;9(2):e1003173. doi: 10.1371/journal.ppat.1003173 (PMC3585129; doi:10.1371/journal.ppat.1003173)
Supplement: Table S1 — Fab crystal structure data collection and refinement statistics. Fab fragments of R880F mAbs 19.3H-L1 (PDB code 4F57) and 19.3H-L3 (PDB code 4F58) were crystallized with the hanging drop method after papain digestion, affinity and size exclusion chromatography purification, and concentration. X-ray diffraction data were collected, processed using HKL2000, and are shown in the top half of the table. Statistics in parentheses in the 19.3H-L1 and 19.3H-L3 columns refer to outer shell resolutions. The structures were refined using COOT and PHENIX and analyzed using ICM; these values are shown in the bottom half of the table. (DOCX) [file ppat.1003173.s003.docx]

**Table S1. Fab crystal structure data collection and refinement statistics.**

|  |  | **19.3H-L1** | **19.3H-L3** |
| --- | --- | --- | --- |
| **Data Collection** | | | |
| Space group |  | C2 | P2_1_ |
| Cell dimensions | a, b, c (Å) | 152.10, 57.46, 60.04 | 73.40, 126.41, 120.08 |
|  | α, β, γ (°) | 90.00, 112.78, 90.00 | 90.00, 90.08, 90.00 |
| Resolution (Å) |  | 1.70 (1.73-1.70) | 2.70 (2.85-2.70) |
| R_merge_ (%) |  | 4.2 (35.5) | 14.8 (49.7) |
| I / σI |  | 31.9 (2.92) | 11.2 (2.5) |
| Completeness (%) |  | 95.8 (81.8) | 99.8 (99.0) |
| Redundancy |  | 3.8 (3.1) | 3.4 (3.1) |
| **Refinement** | | | |
| Resolution (Å) |  | 41.5-1.70 | 46.5-2.5 |
| Number of reflections |  | 50,557 | 75,512 |
| R_work_ / R_free_ |  | 18.65/20.91 | 20.80/25.64 |
| Number of atoms | Protein | 3,296 | 13,078 |
|  | Solvent | 532 | 682 |
| B-factors | Protein | 24.3 | 34.2 |
|  | Solvent | 34.4 | 34.2 |
| R.m.s. deviations | Bond lengths (Å) | 0.0066 | 0.0075 |
|  | Bond angles (°) | 1.114 | 1.165 |
